# Women’s lived social-cultural and clinical experiences and navigation process after stillbirth in Dodoma Region, Tanzania: A phenomenological study

**DOI:** 10.1371/journal.pone.0331319

**Published:** 2025-09-02

**Authors:** Hashim Yatera, Angelina A. Joho, Golden M. Masika

**Affiliations:** Department of Clinical Nursing, School of Nursing and Public Health, The University of Dodoma, Dodoma, Tanzania; Rural Women's Social Education Centre, INDIA

## Abstract

**Background:**

In Tanzania, stillbirth is a public health challenge. The care provided to women after stillbirth does not reflect standards. Little is known on view of the social and clinical human experience surrounding this tragedy. This study explored women lived social-cultural and clinical experiences and the navigation process after stillbirth in Dodoma.

**Method:**

A phenomenological qualitative approach was conducted from February 15, 2024, to May 30, 2024, involving in-depth interviews with 12 informants (postnatal women) who were purposefully selected, achieving data saturation at 9 informants. Data were transcribed verbatim, manifest coded, and analysed using conventional content analysis. Subsequently, sub-categories and main categories were generated. At the theorizing phase, the conceptual interpretation of categories and subcategories were used to generate the conceptual framework that describes the phenomenon of women’s navigation process from the experienced life challenges towards maintaining the overall well-being after stillbirth.

**Results:**

The study identifies six main categories of exposure, each with three subcategories: ① Unsatisfactory medical care (dissatisfaction with medical care, neglected bereavement care, and improper breaking of information); ② Diversity and adversity in cultural practices (dominance of elders’ power, diversity of cultural practice and emotional responses); and ③Diversity in social practices (diversity of social responses towards the news, diversity in social responsibilities and diversity experience after social responses), ④ Meaning constructed after still birth (unpleasant phenomenon of stillbirth, all suffering for nothing, the baby could be saved), ⑤ Mental and physical health challenges (anxiety and depression, post-traumatic stress disorder, and physical burden of still birth), ⑥ Navigating the stillbirth experiences (self-coping, social support and hospital support).

**Conclusion:**

The study found that women who experience stillbirth face significant social, cultural, and psychological challenges that impact their perception and meaning of their world and their mental wellbeing. Ultimately, they invariably navigate to a state of well-being. This highlights the urgent need for culturally sensitive interventions and dedicated healthcare and social support systems to help mothers recover smoothly and swiftly.

## Introduction

Stillbirth is a term that changes according to different settings. WHO defines stillbirth as the death of the fetus after 28 weeks of gestation before delivery or after delivery [1]. Globally, stillbirth accounts for 1.9 million deaths every year means one stillbirth happens every 16 seconds. Sub-Saharan and central Africa, as well as southern Asia, are the leading regions with a high number of stillbirths [1].Women who live in sub-Saharan Africa are eight times more likely to experience stillbirth as compared to High-income countries [2].

Tanzania, as part of sub-Saharan Africa, is among the top ten countries globally with a high number of stillbirths, accounting for 43 out of every thousand live births [1]. However, the global agenda is to reach fewer than twelve stillbirths per thousand live births [3].

Pregnancy is a significant event that has both social and medical implications. Expectant mothers and their communities celebrate this occasion with various social rituals, like baby showers, that help prepare them for motherhood. During pregnancy, parents undergo significant behavioural changes and create a strong emotional bond with their foetus. However, the loss of the foetus can be devastating for them, making it difficult for them to be recognized socially as parents and leading to persistent anxiety and worries that can affect the next pregnancy [4].

Stillbirth can cause psychological distress in mothers, leading to anxiety and depression. These symptoms can appear immediately and can persist for several years, from 7 to 18 years [5]. Additionally, women who experienced stillbirth may have a fear of the potential recurrence even [6] Also, these women may have a fear of their partners, that the cause of the baby loss [7] confusion, denial and disbelief, and anger [8].These mental and physical adverse effects can extend to subsequent pregnancies and parenthood [9].

In Tanzania, intrapartum and postpartum care given to women after and post-stillbirth is suboptimal according to the national standard [10]. National guidelines don’t cover all medical interventions. Medical management lacks crucial psychological treatment, increasing stress for women and families [11]. Healthcare providers have been reported to have inappropriate communication, and in their communication regarding stillbirth, they do not involve the women’s husbands, which in turn causes more psychological effects to the parents [12].

Stillbirth care in low- and middle-income countries often lacks a holistic approach due to insufficient in-depth studies that explore a wide range of cultural diversity [13,14]. Owing to the cultural, social and clinical environment that expectant mothers are subjected to, their lived experiences after they experience stillbirth must be known to help with the planning of cultural, social and clinical interventions [8]. In Tanzania, there is limited literature in this area, and the few available data are in quantitative studies and lack a holistic view of the human experience of this tragedy [2]. Thus, to fill this gap in the scientific body of knowledge, this study explored the lived experience of women who had stillbirths in Dodoma.

## Materials and methods

### Study approach and design

This study utilized a qualitative approach with a hermeneutic phenomenological design, focused on the lived experiences of women who endured a stillbirth. The phenomenological approach emphasized understanding these women’s experiences by deeply exploring their emotions, perceptions, challenges, and coping mechanisms. By prioritizing individual narratives, phenomenology offers rich, detailed insights into how women navigate the intricate interplay of social norms, cultural beliefs, and clinical care following the experience of stillbirth.

The hermeneutic phenomenological approach often aligns well with thematic analysis because both methods aim to uncover deeper meanings and patterns in participants’ lived experiences. Hermeneutic phenomenology focuses on interpreting and understanding the essence of experiences, and thematic analysis provides a structured way to identify, analyze, and report themes within the data. Together, they ensure a rich and meaningful exploration of social, cultural, and clinical experiences.

### Study area

The research was conducted between 15/02/2024 and 30/05/2024 in Dodoma City in Tanzania, the central region. Dodoma is one of Tanzania’s 31 administrative regions and the country’s capital, with a land area of 41311 Km^2^, as well as the location of the legislative assembly. There are multiple hospitals in the region, including a national mental health hospital, a tertiary public hospital (Benjamin Mkapa Hospital), a Dodoma regional referral hospital, and various health centres [15]. Dodoma has a population of 3,085,625 people, of which 35.3% live in urban areas and 64.7% reside in rural regions [16].

### Study population and eligibility criteria

Women residing in Dodoma who have had stillbirths. Women who experienced intrauterine fetal death before the onset of labour and delivered within three months up to one year before the study were included. Women who delivered at home, those whose numbers and addresses were not documented in the hospital data and women who were very sick during the time of study and could not tolerate the interview were excluded.

### Sampling and sample size

Participants were purposefully selected for interviews; the sampling procedure began after obtaining ethical approval from the UDOM ethical committee. The researcher visited Dodoma Regional Referral Hospital to access client information on stillbirths, reviewing the labour record book (MTUHA) for cases between June 2023 and February 2024. Initially, A total of 65 potential participants were identified with a diagnosis of stillbirth, of which 14 lived outside Dodoma, and 13 stillbirths were diagnosed during intrapartum. Ultimately, 38 individuals were included in the study; however, 9 of their phone numbers were not accessed, and 12 of their phone numbers and addresses were not documented. Only 17 participants recorded their phone numbers in the admission book and were called to ask for permission to be enrolled in our study. 3 denied being enrolled, and 2 reported being sick, therefore, 12 participants were interviewed during the data collection. The sample size was 9, determined by the data saturation of the qualitative data.

### Data collection method

#### In-depth interview (IDI).

In-depth interviews were conducted using a semi-structured format, featuring four open-ended questions complemented by probing questions to capture data while allowing flexibility for participants to express themselves fully on their experiences with stillbirth and the coping mechanisms to navigate life’s challenges and maintain overall well-being. Three questions specifically focused on the mothers’ experience of stillbirth, and one question explored their coping mechanisms. The interview for one informant lasted between 45–90 minutes.

#### Data collection tools.

The researcher used a semi-structured interview guide, which had four discussion topics and probes, and the materials were documented in a notebook and recorded using a tape recorder. Before being utilized in an in-depth interview, the interview guide was verified by experts to ensure that all relevant areas were addressed. S1 Appendix.

#### Data collection procedure.

Participants were followed to their respective homes by the researchers, provided with clear information about the study, and asked to sign written consent. In-depth interviews took place in a private place with the presence of 2 researchers and the participant only, the interview lasting from 45 to 90 minutes. Verbal and nonverbal data were recorded by means of audiotape and field notes, respectively.

### Data analysis

The research team transcribed the audio recording verbatim in Swahili and then translated it into English. The transcripts and memos were imported into the qualitative data analysis software ATLAS.ti, version 7.5.7. The data analysis is passed into four main steps: Decontextualization: We thoroughly read and re-read the text, breaking it down into meaningful segments and describing it inductively to uncover both latent and manifest meanings. Recontextualization: The researcher reviewed the material extensively to ensure a comprehensive understanding of relevant meanings. Different meanings were distinguished using coloured text or exclusion. Categorization: Two researchers generated codes whereby the relevant message was highlighted in the text, and succinct sentences were created; they grouped similar codes to form categories and polished the categories to form themes. When differences arose, the researcher engaged in constructive discussions to understand each coder’s perspective and resolved by referring to the data to ensure that the findings achieved key qualitative research requirements of consensus [17]. Compare interpretations and identify overlaps or unique insights.

**Compilation:** The researcher focused on analyzing the data to construct the most plausible explanation, creating a conceptual framework that illustrates the connections among the concepts realistically and practically.

### Rigor

To ensure credibility, in-depth interviews lasting between 30 and 90 minutes were conducted. In addition, during analysis, data from verbatim transcription of the audio records were confirmed with the field notes. To confirm what each participant communicated, paraphrasing techniques were used during data collection to confirm if the data captured reflected the participants’ views, as member checking was not practical. The study tool and report underwent rigorous peer review to ensure that they captured the intended data. Additionally, the interviews were conducted by the investigators who are experts in the field of maternal health. Dependability was ensured through proper documentation of the study protocol, including the coding process (latent and manifest coding by two researchers based on underlying meaning) and procedures followed during the study. To ensure confirmability, participants’ descriptions were paraphrased during interviews and affirmed for accuracy. An audit trail was established through keeping the field notes, audio records, and transcripts for other members of the study to confirm that the process was ensured throughout the study. Transferability was established by providing a rich description of the Dodoma setting where the study was conducted, encompassing cultural, medical, and economic factors linked to women’s experiences.

### Researchers reflexivity

The researchers, both registered nurses, critically examined their experiences and biases regarding stillbirth. To minimize the influence of these biases on the study findings, one researcher was responsible for recording the interviews, and the other took field notes, ensuring an equitable distribution of responsibilities and a systematic approach to bracketing and controlling biases.

### Data triangulation

To ensure data triangulation, interviewed postnatal women come from different socioeconomic backgrounds. We included different methods of data collection in the in-depth interview and documentary review, which offered unique insights. We also cross-check the data by looking for consistent patterns or discrepancies across sources and methods. This strengthens the reliability of the results.

### Ethical consideration and consent to participate

Ethical clearance was granted by the University of Dodoma Institutional Research Review Ethical Committee (IRREC) with ref no. MA.84/261/69/10 on 30/01/2024. The research adhered to the ethical principles outlined in the Declaration of Helsinki (World Medical Association, 2001). Informed consent forms were provided to all participants, outlining the study’s purpose, methods, potential risks, and benefits. Participants were engaged in a comprehensive discussion to ensure their understanding of the research and to address any questions or concerns. Their voluntary participation was emphasized, with the option to withdraw at any time without affecting their care. Measures were implemented to safeguard participant privacy, confidentiality, and anonymity. Personal names were removed from data collection instruments, Unique identification numbers were assigned to participants, and data was stored securely using encryption techniques. Access to the data was restricted to authorized research team members. Regarding the sensitivity of the topic under the study (stillbirth) and to manage the emotional distress of postnatal mothers, the interview was conducted in a safe environment to ensure the interview setting was private, comfortable, and supportive for both the interviewers and the postnatal mother to feel at ease. During the interview, the researchers showed empathy and understanding through active listening. Validate the participant’s emotions without judgment or interruption. Additionally, the scope of the interview was clearly defined, and participants knew they could pause or stop narratives at any time. The participants’ emotional limits were respected.

## Result

### Participants’ social demographic characteristics

Table 1 summarizes the social demographic characteristics of study participants. The study involved 12 participants, all of whom provided a complete response. The average age of the participants was 32.7 ± 5.8 years. The participants’ ages ranged from 24 to 40 years. Most of the women (91.7%) were married and half of them (50%) had completed secondary education, and more than half (58.3%) were self-employed. Additionally, 66.6% had delivered 2–3 times, and nearly half of them (41%) had one living child, while one-third (33.6%) did not have any living children. 16.6% had experienced a stillbirth before the current one, and 33.3% had a history of abortion.

**Table 1:** Social-demographic characteristics of study participants (N = 12).

Variable	Frequency (n)	Percentage (%)
**Age (years)**		
24–30	5	41.7
31–40	7	58.3
**Residence**		
Rural	7	58.3
Urban	5	41.7
**Parity**		
1	3	25.0
2	4	33.3
3	4	33.3
5	1	8.3
**Living children**		
0	4	33.3
1	5	41.7
2	2	16.7
4	1	8.3
**Number of stillbirths**		
1	10	83.3
2	1	8.3
3	1	8.3
**Number of abortions**		
0	8	66.7
1	2	16.7
2	1	8.3
5	1	8.3
**Marital Status**		
Married	11	91.7
Single	1	8.3
**Education Level**		
Primary	3	25.0
Secondary	6	50.0
College/university	3	25
**Occupation**		
Housewife	3	25
Employer	2	16.7
Self-Employer	7	58.3

### Women lived social-cultural and clinical experiences and navigation process after stillbirth

**Fig 1:**
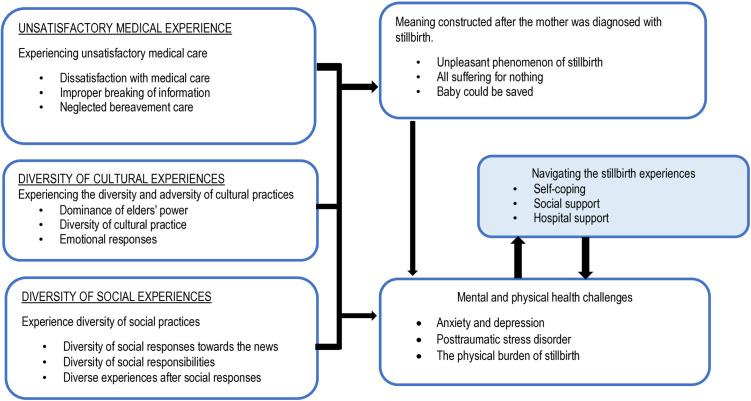
Conceptual interpretation of women lived social-cultural and clinical experiences and navigation process after stillbirth.

This study found the multifaceted experiences of women who have experienced stillbirth in the Dodoma region and their navigation journey towards wellbeing post stillbirth. Analysis of data to understand these multifaceted experiences yielded six categories. The phenomenon highlights three key exposure categories conceptualized as “experiences”: ① Unsatisfactory medical care; ② Diversity and adversity in cultural practices, and ③ Diversity in social practices; two categories representing “adverse outcomes” of exposure categories: ④ Meaning constructed after still birth and ⑤ Mental and physical health challenges; and one category representing ⑥ “Navigation of the adverse outcomes to health and wellbeing”: Fig 1 summarizes the conceptual interpretation of women lived social-cultural and clinical experiences and navigation process after stillbirth.

### Category 1: Unsatisfactory medical experiences

The study found that medical infrastructures are not adequately equipped to prevent stillbirth, even after fetal danger signs have occurred. Many women encounter negative medical experiences, missing crucial interventions that should be provided, such as bereavement care. Collectively, it appears that the medical infrastructure (facility environment, staff, policies, guidelines) is not fully prepared to support women in these situations.

#### Subcategories.

**i. Dissatisfaction with medical care**

First, most women sought medical attention after discovering reduced fetal movement, but they were told to return home after being assessed partially by Pinard fetal scope or Doppler. Unfortunately, the situation continued, and they later found their baby had passed away.

*“He told me, “What are you worried about? The baby is fine, active, and even causing a disturbance. The baby’s heart rate is at 158 beats per minute, which is perfectly normal. Don’t worry, just go back home and wait for the delivery day.” (24-year-old woman)*

Another one added that

*“After two days/the next day, I felt the movements decreasing further, so the following day, I couldn’t feel the baby moving at all. Therefore, I went back to the hospital for another ultrasound check, only to find out that the baby had passed away.” (26-year-old woman)*

Abnormal behaviour from health workers, especially in ultrasound where final diagnosis is obtained.

*“I don’t know if it’s the expertise they are taught or his tactic, but I couldn’t understand. He just left me lying there and walked away and left me and didn’t come back again. I didn’t see them anymore, so I started to feel worried.” (Primigravida)*

During the care process, mothers experienced a sense of isolation due to the lack of specific interventions tailored to this particular group. It appears that health workers were uncertain about the interventions that should have been implemented, ultimately resulting in a gap in care.

*“I couldn’t help but think that perhaps the reason they were skipping me was because I had experienced a loss” (Primigravida)*

Some participants’ impression was that health workers held them accountable for IUFD. This perception sheds light on instances where health workers may, inappropriately shift the focus from counselling to assigning blame to mothers.

*“The blame hurt me, but I didn’t want it to be like that. But it had already happened. Even though they blamed me for being late and for following the advice of older people” (Multigravida)*

**ii. Improper breaking of information**

Many respondents eixpressed dissatisfaction with how the news was delivered, suggesting a lack of proper communication protocols.

*“Honestly, she didn’t use any comforting language. She just kept repeating, your baby has died; he’s passed away. She pounded on the table, saying, “I’m telling you; your baby has died, he’s passed away.” (married woman)*

But others were given the news in ward rounds where everyone listened.

*“I was given the feedback with the doctor who initially examined me, along with a whole team of doctors, students, and fellow patients who were nearby listening” (married woman)*

they were denied the chance to stay with their baby

*“I stretched out my arms and tried to hold her hand and foot, but they told me I couldn’t have her. I only saw her legs and private parts; I couldn’t see her face. I cried uncontrollably” (married woman)*

**iii. Neglected bereavement care**

Majority of women felt that some of these crucial care aspects were missed or neglected, which could have improved their overall experiences.

Some respondents were not allowed to see their stillborn babies.

*“It was a girl, but I didn’t see her. I didn’t see the baby even when I left, I wasn’t even shown my baby” (Married woman)*

The majority of the mothers conveyed a strong feeling that they did not want to be grouped with other women who had live babies.

*“I didn’t want to be mixed up because after that, they took me to the maternity ward. So, that situation was just worsening my condition, especially seeing others breastfeeding and babies crying, and I didn’t have one. I wished I could tell them to move me elsewhere.” (33-year-old woman)*

### Category 2: Diversity of cultural experiences

Cultural experiences emerge from unique practices, beliefs, and traditions that define societies. These varieties place women who have experienced stillbirth on a different spectrum of experiences and evoke different responses.

#### Subcategories.

**i. Dominance of elders’ power**

Elders are entrusted with influential roles in decision-making; However, the outcomes of these decisions can profoundly impact women’s health and, regrettably, may result in cases of intrauterine fetal demise (IUFD).

*“I asked people close to me, saying that the baby hadn’t moved since yesterday and was unusually … I decided to ask an elder. At first, she said it was normal and that sometimes the baby is resting, but it turned out it was not normal.” Prime mother*

Some participants highlighted that it is customary for elder women to be the only individuals permitted to bury stillborn infants. This practice is based on the belief that if younger women were to participate, they may be more prone to experiencing intrauterine fetal demise (IUFD) later in life.

*“She was buried. In our culture, adults, especially mothers, handle the burial because they say the baby didn’t cry” (married woman)*

Participants explained that it is not allowed to name the stillborn.

*“They didn’t give her a name. That idea wasn’t even there because the baby had already passed away, and even giving her a name is something you can’t do because you’ve already prepared for her. Tradition doesn’t allow for that.” (married woman).*

Some participants revealed that their stillborn was buried early, even before they were discharged.

*“They buried him that same night, as I had just had surgery at one o’clock. So, they buried him that very night”* (34-year-old woman)

The majority of the participants revealed that their stillborn was buried in their compounds.

*“At home, we have a farm, so they usually go and bury them in our fields”(multigravida)*

Another one added

*“They just showed me where they had laid her to rest. It’s behind our house, at my father’s place, right here. It’s our tradition because even my husband’s family is a bit far, in Musoma. Normally, I just know they dug a hole to bury her and placed her there.” (multigravida)*

Moreover, Participants explain that they practice religious rituals during burial services, especially those who live in urban areas.

*“They take him, recite prayers over him, wrap him in a white shroud, and bury him with the guidance of an Islamic leader” (38-year-old woman)*

Some women felt that a proper burial ritual reduces emotional stress.

*“He was buried because he was a fully developed human being, not just a miscarried fetus due to bleeding or other complications. He was fully formed, so he couldn’t be discarded or left at the hospital. Leaving him there would have caused more emotional distress.” (multigravida)*

**ii. Diversity of cultural practice**

Following IUFD, most participants discuss a range of cultural guidelines, encompassing traditions and norms that specify the expected conduct for women who have experienced stillbirth.

Participants explained that it is not allowed to name the stillborn.

*“They didn’t give her a name. That idea wasn’t even there because the baby had already passed away, and even giving her a name is something you can’t do because you’ve already prepared for her. Tradition doesn’t allow for that.” (multigravida).*

Some participants revealed that their stillborn was buried early, even before they were discharged.

*“They buried him that same night, as I had just had surgery at one o’clock. So, they buried him that very night” (Primigravida)*

The majority of the participants revealed that their stillborn was buried in their compounds.

*“At home, we have a farm, so they usually go and bury them in our fields”(married women)*

Another one added

*“They just showed me where they had laid her to rest. It’s behind our house, at my father’s place, right here. It’s our tradition because even my husband’s family is a bit far, in Musoma. Normally, I just know they dug a hole to bury her and placed her there.” (Primigravida)*

Moreover, Participants explained that they practice religious rituals during burial services, especially those who live in urban areas.

*“They take him, recite prayers over him, wrap him in a white shroud, and bury him with the guidance of an Islamic leader” (married woman)*

**iii. *Emotional responses***

After exposure to different cultures, the most significant cultural influence often guides the handling of stillborn births, eliciting specific responses from women. Some women felt that a proper burial ritual reduces emotional stress.

*“He was burried because he was a fully developed human being, not just a miscarried fetus due to bleeding or other complications. He was fully formed, so he couldn’t be discarded or left at the hospital. Leaving him there would have caused more emotional distress.”(married woman)*

Also, they felt satisfied with the practice.

*“Preserving him was better, even though it seemed like reopening old wounds. But it’s better knowing that my child is in this place.”(married woman)*

### Category 3: Diversity of social experiences

Pregnancy involves the woman, her family, and society. They all have roles to play from beginning to end. These collective involvements give rise to a diverse array of experiences and responses among women who have undergone the unfortunate event of IUFD.

#### Subcategories.

**i. Diversity of social responses towards the news**

The news of IUFD is not only devastating for the mother but also the family members. Women who have experienced stillbirths may encounter negative responses from family members and society when sharing this unpleasant news.

Participants experienced their families shocked after sharing the news

*“When I told them about the loss, everyone was shocked. They went to inform my mother, who was in the village. The news affected her so much that her blood pressure rose” (Primigravida)*

*“When relatives come to see me, I also cry, and their tears make me start crying again” (Primigravida)*

Some family members were confused and placed into a state of dilemma,

*“That I was told to be admitted and also that the baby had passed away in the womb. They couldn’t believe it and said, how is it possible for the baby to pass away in the womb?” (Primigravida)*

These questions continue and create a state of anxiety which is not hidden even from the pregnant woman who had IUFD.

*“So, when she heard that the baby had passed away in the womb, she wondered how the baby would come out or if I would die also.” (Primigravida)*

**ii. Diversity of social responsibilities**

Following an intrauterine fetal demise (IUFD), families and their communities have a responsibility to ensure the well-being of the mother. This involves providing supportive care to help the woman cope with the effects of the stillbirth.

*“So, they had to start staying with me and bring me back to health” (Single mother)*

*“I was assigned a relative to stay with me. I had a relative with me at that time, and it helped me a lot” (Single mother)*

Additionally, assistance is provided throughout the postnatal period, ensuring that individuals receive the same level of care as those who have live babies.

*“I was taken care for a whole month because my sister in-law also left home and stayed for a whole month” (Primigravida)*

*“All the postnatal care was good. For example, my sister would massage me with warm water every day. She would prepare meals for me every morning, and whenever I needed assistance, she was there to provide it.” (Primigravida)*

Family members and their society must spread the news to the community.

*“Once one person knew, the others would find out too as they would spread the news” (Multigravida)*

Furthermore, they must bury the stillborn.

*“I have never been taken to do so because whenever such things happen, I am usually in the hospital. So, relatives, gravediggers, they just go and bury as usual” (Primigravida)*

**iii. Diversity of experiences after social responses**

Social responses and responsibilities provoke a variety of maternal experiences, which can be positive or negative. Mothers experience profound sadness as a result of the social condolences they receive, perceiving these expressions of sympathy as persistent reminders of their loss.

*“So, when people came to see me there, I felt bad. They came to offer me condolences, but I wished they had found me busy breastfeeding the baby or receiving the child” (Single mother)*

Another one added

*“They told me, I’m sorry, that’s life, everyone has their problems, deep down, it hurt me, even though you accepted it.”(Married woman)*

Others had a sense of failure to meet family expectations, and it induced emotional pain

*“It was painful because when a pregnant woman goes to the hospital, everyone expects her to return with a baby, but in my case, I returned empty-handed” (married woman)*

*“You must feel pain because this sympathy is given to me because I lost a child, but if I had a child, I would have been congratulated” (married woman)*

Overall, they felt satisfied with the care they had received during the postnatal period

*“The care was good; my mother went to great lengths to provide me with maternal care, even though she already knew the baby had passed away.” (24-year-old woman)*

*“Nothing was lacking. I received excellent care. I was bathed and attended to just like a patient.” (24-year-old woman)*

### Category 4: Meaning constructed stillbirth

Participants, after undergoing various events and experiences of care, have been able to extract their experiences into a concise meaning that effectively encapsulates the phenomenon from a comprehensive perspective.

#### Subcategories.

**i. Unpleasant phenomenon of stillbirth**

The occurrence of fetal death is a devastating experience for parents and family, inducing a variety of responses that are unpleasant for any human being to endure. Some participants revealed that it is a painful event

*“You know, delivering a baby who has already passed away, it was painful, but I was praying to God a lot. I just wanted to come out of it safely.”* (P2)

Some felt unpleasant experiences, such that no words could be used to explain the phenomenon to other people.

*“Yeah, in those moments, it’s like... I mean, it’s hard to find the right words sometimes. There are no words, and it was tough.”*

Following the intrauterine fetal demise (IUFD) and delivery, they experienced profound loneliness as the previously established bond with the fetus abruptly disappeared.

*“After he passed away in the womb, I no longer felt him playing, and it made me feel lonely and terrible” (P3)*

Some experienced loneliness after delivery.

*“It felt like I had experienced loneliness; I was separated from my child. You know, being with a creature for nine months, I had already grown accustomed to it.” (P1)*

**ii. All suffering for nothing**

They endure labour pain and provide care only to experience the tragic outcome of a stillborn birth. This situation can lead them to feel that all the suffering was for nothing.

*“My feelings during labour were of intense pain because I knew that I would deliver but not have a baby” (Primigravida)*

**iii. Baby could have been saved**

A woman reflects on the past and tries to figure out what could have been done to save the life of her fetus

*“It’s hard to forget the experience during the 37th week, especially with the mistreatment you endured. It seems they failed to provide you with proper care, despite having the capability to save the baby’s life.” (Primigravida)*

For them, it implies that they are simply an unlucky victim of stillbirth.

*“But if you are alone with a stillborn in a room, you will feel like the whole hospital is mourning only your loss” (Primigravida)*

### Category 5: Mental and physical health, challenges

Mental challenges are dominant, with effects that start from anxiety, depression, and post-traumatic syndrome.

#### Subcategories.

**i. Anxiety and depression**

Anxiety began immediately after noticing reduced fetal movement and subsequently intensified over time.

*“When I drank my tea, I expected him to move, but he didn’t. I started crying right there at home because his lack of movement felt unusual. The fact that he wasn’t moving, something that was usually routine, deeply saddened me.” (Multigravida)*

During ultrasound diagnoses, the behaviour of healthcare professionals can significantly exacerbate feelings of anxiety in patients.

*“But this time, it appeared different. As the doctor examined it for the fourth time, I felt a surge of anxiety and prayed for guidance. It was as if something was being communicated to me through their whispering.” (Married women)*

Anxiety continues into the postnatal period, where they have felt fear that they might lose their husbands or be mistreated.

*“I wondered how it would be if my partner demanded a baby and I didn’t fulfil it. Would he accept me, or would he seek someone else? Would he see me as unlucky and mistreat me?”(Married woman)*

Others thought it would be better to die with their fetus because they felt worthless*.*

*“I felt awful and even thought it would have been better if I had left with the baby. I felt worthless” (married woman)*

Moreover, peace was disturbed after knowing they had a dead fetus in their womb.

*“When I sleep, I don’t feel at peace. It’s like he’s following me, almost like someone is stalking me. When I turn, it feels like he’s still there.” (married woman)*

Additionally, individuals instructed to await for the onset of labour reported experiencing disturbances in their sleep and an inability to sleep alone.

*“When they told me to sleep and wait for the onset of labour so they could figure out how to assist me, it added to my pain because I felt I couldn’t even sleep alone” (26 years old woman)*

Excessive crying has been observed among the majority of participants.

*“I used to wake up crying in the morning, cry during the day, and cry at night” (Primigravida)*

Some women report experiencing a period during which they encounter overthinking.

*“I was constantly wondering, asking myself, why? why? why? This self-questioning caused me trouble at home (she cries).” (Primigravida)*

**ii. Posttraumatic stress disorder**

Symptoms of Postpartum post-traumatic stress disorder (PTSD) may persist after childbirth through the postpartum period.

At this period, women report experiencing flashbacks

*“You see, so when you stay alone for a long time, that image starts to bother you. So that image kept coming back... oh, quite often” (Multigravida)*

Others have recurrent distress dreams about the event

*“I mean, all the time I would dream or verbalize that thing. Such dreams about the baby often occurred, but now I’m fine” (Multigravida)*

Additionally, other participants experienced a state of self-taking

*“I’m grateful that I’m okay even though it troubled me. I used to talk with myself constantly.” (married woman)*

**iii. The physical burden of stillbirth**

The physical burden begins with fetal demise and persists after birth. Pregnant individuals experience a sensation of added weight, which can affect mobility by putting pressure on the pelvis and creating numbness in the legs.

*“Weight of the pregnancy increased, and the belly became bigger, as if it was filled with gas” (Multigravida)*

Another one added that

*“I began to feel different, the sensation with the baby who had passed away felt heavier, like I was carrying a heavy ball. With a baby who’s alive, their movements aren’t as heavy. So, when I slept from Thursday to Friday morning, the baby seemed to be pressing heavily on one side, to the point where my legs felt numb, unlike previous days.” (Married woman)*

### Category 6: Navigating the stillbirth experiences

Following a stillbirth, women have a responsibility to implement strategies aimed at managing stress, challenges, and difficulties. These mechanisms can be reinforced through self-care practices or external support from family, healthcare professionals, and society.

#### Subcategories.

**i. Self-coping strategies**

Upon receiving the news, individuals initially strive to find ways to calm themselves to effectively deal with the situation and minimize further adverse effects. This behavioral process is commonly referred to as resilience.

*“ I thank God, I had just gotten the courage and strength. I got it because the pressure was already high, so I said to myself, here, if I continue to bring more fear, I might end up causing myself even more problems.” (Multigravida)*

After delivery, the environment also plays a significant role in helping individuals cope. Those who were placed in a separate room within the regular group or given a private room found it easier to cope and acknowledged the benefit of being alone. This facilitated the opportunity to seek companionship, which ultimately aided them through the grieving process.

*“Honestly, being alone in the ward also helped me. I think if I had been placed in a ward with other mothers and their babies, I might have experienced more pain.” (married woman)*

Upon realizing that they were not alone in experiencing such events, individuals experienced a profound sense of relief and found support for their coping journey. They gained valuable insights by learning about different experiences and how others overcame similar challenges.

*“Then you realize that it’s not just you, but a challenge for many others as well. It’s not only you experiencing this, so it’s important to just thank God” (married woman)*

Another one added

*“We were many, and seeing others going through similar struggles brought comfort. It made me feel like I wasn’t alone.” (married woman)*

Spiritual meditations, such as prayer, were among the strategies used to cope with the stress of the event at various stages. This ranged from the initial news of IUFD to later stages of life.

*“The main thing is to thank God. When you find yourself in that situation, you turn to prayer and God grants you strength to continue with life.” (married woman)*

Engaging in a variety of reproductive activities was observed as a coping mechanism by some women, providing them with a sense that life should continue.

*“I had to stand up for myself that there are other things I need to do so that life can continue, I went out and went to my businesses, going to church. It helped me a lot.” (married woman)*

**ii. Social support for coping strategies**

The involvement of family, peers, and social support is crucial in facilitating coping mechanisms. Women experienced relief from the burden of the stillbirth event when these figures were integrated into their care.

*“My friends who were also admitted came to visit me, so I felt hopeful because even in the ward where I was admitted, others were facing similar challenges like mine. We talked, and they comforted me” (married woman)*

Another one added

*“It’s better if they stay in their rooms. There, they can comfort each other, cry together, pray together, and console one another. Eventually, they will all see that these are normal things to go through.” (Multigravida)*

Furthermore, support from family plays a crucial role in coping during hospitalized and after discharged

*“When you’re in the hospital, you know your relatives will come, bring you tea, and someone will bring you porridge, at least to comfort you. They won’t leave you alone.” (Primigravida).*

*“At home, everyone around us is a great source of comfort. I felt really good; they offer condolences and give advice” (Multigravida)*

**iii. Healthworks support for coping strategies**

Accepting reality is an effective coping mechanism employed by health workers to support women who have experienced stillbirth. This approach aided in addressing the mental challenges arising from the traumatic event and is often utilized in counseling sessions to assist in the facilitation of the healing process.

*“They were very close to me, and I was also given counselling. It helped me a lot, and a doctor also came to counsel me. So, I accepted the situation.” (married woman)*

## Discussion

The study explored the lived experience of women who had stillbirths. The study reviewed key findings of women’s experiences after stillbirth, which include unsatisfactory medical care, diversity and adversity in cultural practices, diversity insocial practices, meaning constructed after stillbirth, mental and physical health challenges and navigation of the adverse outcomes to health and wellbeing.

Mothers who reported experiencing unsatisfactory medical care cited dissatisfaction with medical services, improper handling of information, and neglected bereavement care. This aligns with studies conducted globally; for instance, a systematic review and meta-summary by Shakespeare et al [18] indicated that many women expressed dissatisfaction with the quality of care they received due to a lack of recognition following stillbirth, which led to negative feelings such as stigmatization, blame, devaluation, and loss of social status. Another study in Afghanistan noted that women were not allowed to decide whether to see and hold their stillborn babies, which was interpreted as regret [19]. Holding and seeing the stillborn is vital for mothers and parents who have experienced the loss of their newborn, as it helps mitigate psychological effects and fosters memory-making. Some parents may take photographs of their baby as tangible proof of the child’s existence [7]. Dissatisfaction was also reported in the study conducted in India, which reported that providers were insensitive, negligent and disrespectful of mothers who suffered stillbirths, which aggravated the feelings of grief and guilt [20].

Regarding the improper breaking of information about stillbirth, it causes negative feelings. Various studies reported similar events, such as a study done by Carol et al [7], verbal communication deteriorated, unsatisfactory medical care affected women’s satisfaction and caused a sense of isolation. Another study conducted by Shakespeare et al [18] women who experienced stillbirth wished to have received information in their local language could have improved their grief, fear of delivery of stillbirth, fear of their health and could have controlled the grieving process. Lack of good communication was also reported in India, where women were not informed of the cause of stillbirth [20].

Evidence indicates that women who experienced stillbirths received inadequate medical treatment during pregnancy and delivery, perceiving that essential aspects of care were overlooked and that grief support was insufficiently integrated. Bereavement care aids women and families in managing stress and preventing mental health issues [6]. Denying women time with their stillborn contradicts the continuity bonding theory, which emphasises maintaining a relationship with the stillborn. Establishing a positive closeness, connection, and dialogue with the stillborn may alleviate mental health issues and promote effective coping strategies [21]. New guidelines, standard operating procedures, and policies are necessary for the integration of care for mothers who have had stillbirth and their families. These should address deficiencies in care practices.

Stillbirth mothers navigate a complex network of medical and social dynamics that impact their grief. Various responses occur in this complex situation. King et al [22] observed that many women relate stillbirth with deep sorrow and struggle to express their emotional suffering. Women who have suffered hardships before stillbirth, such as poor care and labour discomfort, may conclude that their suffering was pointless [22]. The findings support narrative theory, which states that people create narratives shaped by their life experiences to make sense of traumatic events. Different perceptions can affect an individual’s emotions and coping processes [23].

Due to stillbirth experiences, some mothers believe their baby may have been rescued by suitable interventions. Studies show that community education can prevent stillbirth and prompt medical measures, especially when antenatal care shows diminished fetal movement [20,24].

Society regulates behaviour by suggesting what they should and shouldn’t do and how to help them based on social and cultural variety. Most informants live in cities and forego cultural practices and religion. This power shift also affects mothers’ behavior and beliefs, attributing stillbirth to God, according to Allahdadian & Irajpour [25]. Family prayer strengthens coping mechanisms. Studies show that integrating spiritual needs into medical care improves bereavement care and family recovery [26].

The study found that certain cultures view stillbirth as quiet. Mothers cannot attend the burial ritual, which is for elder women. The stillbirth is not reported, and no burial or public gathering is held, as is common with infant deaths. Instead, stillbirth is typically ignored. Tanzania, Uganda, and Taiwan had similar procedures [27,28].

After stillbirth, family and community support helped cope with loss and sadness. Cultural views about stillbirth caused stress, fear, and stigma, especially for women. These traditions sometimes prevented parents from holding and seeing their stillborn, discussing the loss, creating memories, or participating in the funeral. Parents struggled to grieve and adapt because to the difficult balance between personal demands and communal expectations [29].

The current study supports the continuum bonding hypothesis by showing that mother-family social and spiritual events improve their well-being. These experiences reinforce good memories of the stillborn baby’s afterlife well-being. However, negative spiritual experiences relating to the deceased may cause confusing emotions and thoughts for women and their families. Some consider social memory-making illogical and a failure to cope [21].

Comprehensive and culturally responsive interventions to destigmatize stillbirths are needed to meet community needs. These programs should also help parents grieve culturally and individually appropriately.

Mental health issues commonly arise when a mother becomes aware of decreasing fetal movement, especially if it was mismanaged throughout diagnosis, hospitalization, and discharge.

A comprehensive review examined short- and long-term sadness, anxiety, and PTSD in parents of stillborns from diverse countries. The review found consistency across regions [5]. Kübler-Ross’ 1969 five-stage grief theory matches the data. After learning of death, people go through denial, anger, bargaining, depression, and acceptance. Although the stages may not occur sequentially, the people involved may follow distinct patterns and move at different speeds [30].

However, existing mental difficulties and a lack of interventions to address and manage them put mothers who had stillbirth and their families at risk of emotional exhaustion. They may struggle to manage due to emotional overwhelm [9].This call for the needs of mental and physical health interventions, especially postnatally (stillbirth aftercare).

Positive coping skills can help mothers who have had a stillbirth avoid health issues. Internal and external variables like healthcare providers, peers, family, and society might promote these coping techniques. The interaction of these internal and environmental elements might lead to negative or positive coping mechanisms.

Stillbirth’s emotional, social, and economic effects force women to change, often without notice. Thus, healthcare professionals must recognise this key stage and offer constructive coping techniques [31]. Findings allies with Resilience theory, which addresses trauma recovery, and healing requires personal, social, familial, and institutional safety nets. These factors help people handle life stress [32].

Women want different, more integrated care than live birth mothers. They stressed the importance of bereavement counselling, professional help for stillbirth mothers, and social and family support. Provide comprehensive care for all mothers who have had stillbirths to reduce mental health issues and promote good coping. Previous investigations [12,33–35] support these findings.

We must recognize that women and their families may want different grief support. Some favor photography or cultural practises, while others prefer other methods [36]. Therefore, a holistic approach with a variety of options is essential to allow women to pick the care that best suits them.

### Limitations

Even though researchers were aware of their own experiences and biases that may originate from some having experienced stillbirth directly or indirectly, and or participating in medical care as clinical experts, there may be some instances where the women’s accounts of their experiences are exaggerated or understated. Nevertheless, the researchers tried their best to bracket their feelings and let the findings reflect what was communicated by the participants. Another limitation may be related to the emotional and psychological distress of participants. Women recalling and discussing their experiences with stillbirth may experience emotional distress, which potentially may affect the depth and accuracy of their accounts. Some participants may refuse to share some information due to emotional pain, stigma, or cultural barriers. In addition, women may have difficulties recalling all the past information and presenting it accurately, also affecting the accuracy and completeness of the information. This may have affected the depth or accuracy of the women’s experiences about stillbirth and their navigation to maintaining their wellbeing.

## Conclusion

The study found that women who experience stillbirth face significant social, cultural, and psychological challenges. It emphasises the urgent need for culturally sensitive interventions and support systems to help mothers process grief and navigate their healing journey. Furthermore, improving the quality of antenatal care and emergency obstetric services is essential to prevent such losses. Addressing the psychological needs of these women remains critical. Additionally, the support of family members, partners, society, and religious communities plays a crucial role in aiding their recovery through the provision of psychological care.

## Supporting information

S1 Appendix Interview guide on exploring the experience of stillbirth among postnatal women. (DOCX)

S1 Data The data reflects participants’ social-cultural and clinical experiences, as well as their navigation processes within the healthcare system. (DOCX)
