# Supplementary material for: Women’s lived social-cultural and clinical experiences and navigation process after stillbirth in Dodoma Region, Tanzania: A phenomenological study
Source: PLoS One. 2025 Sep 2;20(9):e0331319. doi: 10.1371/journal.pone.0331319 (PMC12404377; doi:10.1371/journal.pone.0331319)
Supplement: S1 Appendix — (DOCX) [file pone.0331319.s001.docx]

**Appendix 1**

**Interview guide: Exploring the experience of stillbirth among postnatal women**

1. **Please share your experience when you discovered that you had intrauterine fetal death**

**Probing: Can you tell me more about**

- 1. Did you note something wrong before going to the hospital?
  2. How was information provided?
  3. How did you feel after given that information?
  4. How did the family take that news?
  5. Do you feel you got enough support from family and health care providers?

1. **Please share your experience with family response after discovering that you had an intrauterine fetal death.**

**Probing: Can you tell me more about**

- 1. Were there any cultural issues?

1. **Please share your experience when you received care after discovering that you had an intrauterine fetal death.**

**Probing: Can you tell me more about**

- 1. the care you received before and after childbirth
  2. What is your experience regarding the care?
  3. Would you wish to experience different care?

1. **Tell me how you cope after experiencing a stillbirth**

**Probing: Can you tell me more about**

- 1. Did you get any support from your family, community, or hospital?
