# Supplementary material for: Women’s lived social-cultural and clinical experiences and navigation process after stillbirth in Dodoma Region, Tanzania: A phenomenological study
Source: PLoS One. 2025 Sep 2;20(9):e0331319. doi: 10.1371/journal.pone.0331319 (PMC12404377; doi:10.1371/journal.pone.0331319)
Supplement: S1 Data — (DOCX) [file pone.0331319.s002.docx]

**Transcripts**

| Participant one |  | |
| --- | --- | --- |
| Yes, after we got it, we were happy because we had expected it. | | |
| However, after having it, it started to bother me. I had severe abdominal pain. | | |
| I went to the hospital and they told me it was probably just the usual pregnancy pains, so they didn't give me anything. I went home and then went to the pharmacy, and the pharmacist gave me some pills that they said would help with the cutting pains | | |
| At just under eight months, my baby stopped playing. I went to the hospital and had an ultrasound, and they told me the baby was fine and there were no problems. I went home, but the same thing continued - the baby wasn't playing, just moving around a little. So, I went back to the hospital, and they examined me and said the baby was fine, but the nurse told me, 'I don't know if there's too much amniotic fluid or what.' I didn't understand her, and she told me to wait until Wednesday when I could see the doctor. | | |
| When I arrived, they did some tests first and couldn't find the baby's heartbeat. They did another ultrasound and asked me, 'When was your last ultrasound?' I told them, 'Wednesday.' They asked, 'What were you told?' I said, 'I was just told that the baby is fine.' They then told me, 'The baby has died in the womb three days ago.'" | | |
| After the ultrasound, the doctor came in and told me, 'Unfortunately, your baby has passed away. Therefore, you will need to have a C-section to deliver the baby. A vaginal delivery is no longer possible. | | |
| Oh, they just told me that my baby has died in the womb. They didn't tell me what the cause was at all | | |
| How much did he weigh? They didn't tell me, but they were just talking to themselves. I don't know, three kilos, I don't know how much. He was just big.  I wasn't even shown. | | |
| In short, it hurt me, yes | | |
| Others have their children, while you are left in pain. Yes, you've lost a child again." | | |
| In truth, on the other hand, I was grateful. Perhaps if I had seen him, it would have hurt me even more. | | |
| I really don't know the reason, I don't know until now. I wish I knew, that's what brought me here, I wish I knew the reason | | |
| I told him, but he didn't believe me. | | |
| The baby had already been dead in the womb for three days and had started to decompose. They buried him that same night, as I had just had surgery at one o'clock. So they buried him that very night. | | |
| There was no funeral, they just buried him. | | |
| The home care services I received were the same as what other parents got | | |
| It's just that, well, my loved ones to be around me, just to be there to comfort me | |  |
| On that ultrasound, because of how I was feeling pain, they told me it was just because of that condition (hmm),,, maybe, but maybe if I would have felt that maybe they would have discovered it earlier and helped me. | | |
| I needed it, but unfortunately it did not happen. However, I gave birth at eight months.  I was truly happy, After that, I received it with joy and continued to attend the clinic regularly every month for check-ups. | | |
| But the last time, eight months before attending the clinic, I noticed these signs because the baby was moving. Then, after a while, the baby stopped moving. On the first day and the second day, I asked people close to me, saying that the baby hadn't moved since yesterday and was unusually still. I decided to ask my mother. At first, she said it was normal and that sometimes the baby is resting, but it turned out it was not normal | | |
| So, I delayed going to the hospital because I believed what they were telling me. Since they were older people, I thought they knew better, so I kept delaying. When I went for an ultrasound, I was told that the baby had died in the womb, so I delayed getting to the hospital quickly. The baby used to move before, so I didn’t know what caused it, but the doctors here told me that the baby had pressure, possibly on the head. That’s what I understood from what they told me | | |
| At the ultrasound, they explained the steps for removing the baby from the womb. So, right there, they told me, and my husband was also with me. They told me I had lost the baby. | | |
| I felt really bad, I couldn't believe it. Even today, I still feel like the baby is calm or sometimes babies calm down, so I was shocked to be told that the baby had died in the womb. I didn't feel well. | | |
| He took it badly (husband). | | |
| I was very scared... I mean, I was scared to hear that the baby had died in the womb. I was scared for the first time, hmm. | | |
| I just felt bad about losing the baby, and the fact that the baby had died in the womb was difficult for me to accept. All that was left was to remove the baby, which wasn't easy for me. I took it badly. | | |
| When the baby came out, the doctor took the baby first and left me there alone. I didn't know where he was taken. | | |
| Oh, it was just a girl, but I didn't see her. I didn't see the baby even when I left. Oh, I just didn't want to see her. The situation here, my head wasn't right. | | |
| I felt that if I saw her, it would affect me even more. I was already feeling overwhelmed at that moment, so I didn't want to see her. | | |
| After he finished delivering the baby, he covered her and left with her. But he asked me if I wanted to see her. | | |
| After that, when I was there, they took me to a resting area, but I got a fever, and I didn't know where it came from. I was shaking so much that I lost consciousness. I didn't know what the problem was because it happened after leaving the delivery room. I don't know if it was the cold because they left me very exposed, so I felt cold and shivered a lot. I even called my parents to come back to the hospital because they had already left. Is like I was losing my mind based on how they were explaining it. | | |
| I think the problem was psychological. It was just me; I had too many thoughts because I really wanted to be a mother. | | |
| Oh, I really didn't want to be mixed up, because after that, they took me to the maternity ward. So, that situation was just worsening my condition, especially seeing others breastfeeding, babies crying, and I didn't have one. I wished I could tell them to move me elsewhere. | | |
| So, the blame arose from the fact that we were late to the hospital. When we went for the ultrasound to see if the baby was safe, that's when we were told the unfortunate news that the baby had already passed away. That's where the blame started, directed towards the doctors | | |
| The blame hurt me, but I didn't want it to be like that. But it had already happened. Even though they blamed me for being late and for following the advice of older people, I realized that I should seek medical advice directly from doctors because they know better. | | |
| Yes, she was buried. In our culture, adults, especially mothers, handle the burial because they say the baby didn't cry. | | |
| They just showed me where they had laid her to rest. It's behind our house, at my father's place, right here. It's our tradition because even my husband's family is a bit far, in Musoma. Normally, I just know they dug a hole to bury her and placed her there. | | |
| They didn't give her a name. Actually, that idea wasn't even there because the baby had already passed away, and even giving her a name is something you can't do because you've already prepared for her. Tradition doesn't allow for that. | | |
| I wouldn't have wanted her to be given a name because when I remember the name and the baby isn't here, its better if it stays that way because she's not here. | | |
| When I gave birth? After that, I felt like I had a fever, so I had to stay in the hospital for about a week and a few days, maybe two days. When I finished the drip and medication, that's when I was discharged because they saw that I was doing well, so I was given permission to leave. | | |
| Currently, I'm grateful that I'm okay even though it troubled me. I used to verbalize constantly. I mean, all the time I would dream or verbalize that thing. Such dreams about the baby often occurred, but now I'm fine. | | |
| At home, everyone around us is a great source of comfort. I felt really good; they offer condolences and give advice here and there. Hmm, even when I was leaving the hospital, people were genuinely coming to see us. | | |
| I found that there was a good space for me to stay there. It was decided by family members that I should go to my grandmother's place. The environment, let's say, was comfortable for me to rest. There was supervision, someone to watch over me and perhaps attend to my needs. However, they are often at work. That's why it wasn't easy for me to go there. They are often at work. That's why it wasn't easy for me to go there. | | |
| And often they would ask me about everything that happened, even the doctors themselves couldn't believe why it happened like that. They often asked me what happened. | | |
| **Participants 3** | | |
| I struggled and went to many hospitals looking for a problem but found none. So, when this happened, I found myself in the first and second month without believing it. I said it might be true and asked my husband. He said, No, maybe you've just missed your period; go get tested, go to the hospital.' I didn't go to the hospital; instead, I bought a pregnancy test. I tested myself and found that I was indeed pregnant. I couldn't believe my eyes because I realized it is possible. Don't lose hope, because getting married and staying for many years without a child, like me for over 18 years, can be difficult. | | |
| Mmmmh, you know there are challenges with relatives and in-laws, and everything becomes a real test. Sometimes you completely lose hope. When I saw it happen, I said, 'God, you have remembered me.' Truly, I knelt down, cried, and thanked God. | | |
| Upon hearing about my pregnancy? Oh, everyone was overjoyed; they all rejoiced, especially my mother. She would sit and ponder, thinking, Does this mean my daughter, after all these years of marriage, is finally going to have a child? She kept telling me, My child, come back home now. What are you doing there? I would respond, When I face God, I won't be asked how many children I bore, but rather what I did with my life on Earth. So, I trusted in God because I am a Christian. I believed in God through all of this. On the other side, some people began saying that the Chaga people don't like having children; they prefer living leisurely. You could marry into other tribes and find differences. They started saying that Chagas don't like having children; they are afraid of aging. But that wasn't the case because no one enters into marriage without wanting to see the fruits of it. No one desires such things. We desire success, to have children to play with at home. It's not fulfilling to be alone like this. Hmm. | | |
| I began feeling unwell; my stomach was hurting, I remember it was hurting so much that I was sweating profusely. My heart was racing, and I wondered why this was happening, especially since I wasn't due to give birth for several more months. I thought, What's wrong? I decided to call one of my relatives who had a car. I told him I wasn't feeling well. Fortunately, I had already prepared everything, even put on suitable clothes. I told the driver to start the car, and we rushed to the hospital. When we arrived, my blood pressure was 290/290. They told me, No, this blood pressure is extremely dangerous; it could lead to eclampsia. I am truly grateful to God that they took good care of me. | | |
| However, after continuing to stay there, they noticed irregularities in the baby's heartbeat. They took me for an ultrasound and realized that something was not right. They decided to intervene. | | |
| Upon arriving and being taken to the ultrasound room, they observed that there was no amniotic fluid surrounding the baby in the womb. | | |
| We reached there on the first day and stayed, but during the night, they informed me that the baby had passed away. | | |
| After the ultrasound, they were discussing among themselves, pointing at different things on the screen. I asked them what was happening, wanting to know the truth. | | |
| I felt my anxiety increase as they whispered among themselves, wondering why they were doing so and fearing the worst. | | |
| When they finally spoke, it was after a long time. They took quite a while, examining the ultrasound multiple times. When the doctor looked at it for the fourth time, I was startled. I wondered why the first and second times seemed normal, but this time, it appeared different. As the doctor examined it for the fourth time, I felt a surge of anxiety and prayed for guidance. It was as if something was being communicated to me through their whispering. However, later, they reassured me not to worry and took me back to the waiting room.Top of FormBottom of Form | | |
| At that moment, they hadn't informed me yet, but I sensed that something was wrong, especially with their prolonged discussion and examinations. It felt like they were withholding information. Later, they told me to stay calm first. | | |
| They eventually informed me that the baby had passed away in the womb. I felt awful and even thought it would have been better if I had died with the baby. I felt worthless. But later, I prayed to God, asking for forgiveness because I had hoped to become a mother, as I longed to be called mom. | | |
| I said to God, "Help me, all the insults are gone now. I'm going to be called 'mom' again, even though the baby passed away in the womb. I started to wonder, "How does a baby come out after passing away in the womb?" This raised many questions for me. | | |
| I don't know if the baby passed away in the evening and I spent the entire night with them until later, when I eventually gave birth that night. | | |
| I used to spend time with him myself, and when I sleep, I don't feel at peace. It's like he's following me, almost like someone stalking me. When I turn, it feels like he's still there. When the baby was alive, sometimes he would kick and move around, causing me so much pain. Even now, I still feel upset about it | | |
| It was different when he was healthy. I loved calling him "gift" and would pray for him every morning, saying, My child, gift. I'd wake up and pray for him and myself, wondering why I didn't hear him playing. It was as if I had built a routine, expecting him to kick or move around. After he passed away in the womb, I no longer felt him playing, and it made me feel very bad. | | |
| When they heard about my pregnancy, they started preparing. They went to buy clothes and everything else. When I told them about the loss, everyone was shocked.They went to inform my mother, who was in the village. The news affected her so much that her blood pressure rose, and she had to be taken to the hospital. She remained unconscious for two days before finally regaining consciousness. She had already anticipated seeing her grandchild from me, so when she heard that the baby had passed away in the womb, she wondered how the baby would come out or if she herself would die. | | |
| I prayed to God, telling Him that I don't rely on humans; I rely on Him alone. If the baby comes out, it's by His will; if not, it's still by His will. I wished for the baby to come out because he had already passed away, and I wondered how long he would stay inside. We thanked God because we didn't rely on cultural customs; we relied on prayer. Considering that God had saved me during my time in the hospital, we prayed and trusted in Him. | | |
| They were all wondering how the baby would come out. Everyone was preoccupied with thoughts about it. That's why my mother, even to this day, calls me every morning just to confirm if I am safe. She can't believe that I have come out safely. | | |
| When I gave birth, they told me to look at the baby's gender. I looked and tears started flowing because I had never seen my child before. I did not got the chancer to breastfeed him, I felt terrible. | | |
| In that moment, the doctor really hurt me. They made incisions both internally and externally. Internally, they stitched me up with five stitches, and externally, I received another five stitches. While I was pushing, the area torned, and the baby came out very quickly, almost slipping out. They didn't support me properly; no one held perinium me during the process. | | |
| After they tied the umbilical cord, the doctor told me to check the baby's gender. When I looked and saw it was a girl, they said, I'm so sorry, mama. That moment, the image of that scene is something I don't think will ever leave my mind. I stretched out my arms and tried to hold her hand and foot, but they told me I couldn't. I only saw her legs and private parts; I couldn't see her face. I cried uncontrollably, and they began to feel sorry for me. | | |
| After I was returned to the parents' room, in our room, there were seven beds. Out of the seven beds, two were occupied, including mine. | | |
| Every time they were called to breastfeed and left me alone, I felt intense pain. It seemed like each event was causing me significant distress. That was the major challenge I faced. | | |
| It would indeed be beneficial if people experiencing challenges like mine were placed in their own designated area. The environment they create in there can be quite distressing. You can see how it adds to the emotional burden, contributing to increased pressure as we contemplate our situations. | | |
| People facing such challenges should indeed be placed in their own designated area where they can support each other without the presence of those with children. This would provide a supportive environment conducive to healing and emotional comfort. | | |
| They said they were giving us three days, implying that if we were unable to collect the baby within that timeframe, the child would undergo incineration along with other birthing waste. This continued to perplex me. | | |
| I called the baby's father, who was traveling, but thankfully, he caught a flight and arrived the next morning. As soon as he got there, he reached out to the local community members because I couldn't go, and together they found a place for burial. | | |
| The only memory I have left is burying my child. I buried her in one cloth, and I kept another one, vowing not to lose it. It's a reminder of burying my first child. | | |
| I asked the baby's father to take a picture, but he said it hurt him deeply, so he couldn't. I suggested my sister, but even she hesitated, fearing to do so even in the mortuary. It would have helped me emotionally to see, as it would confirm that I had indeed given birth. | | |
| They were afraid to touch the baby. However, the baby's father gathered the courage to hold the child and take them to the car themselves. | | |
| I really wanted to go to the burial, but I still had some physical issues, and I hadn't fully recovered. Walking was difficult, and even sitting was challenging for me at that time. | | |
| I asked my father-in-law to take me to the grave where the child was buried so I could see it. | | |
| I looked at him, thinking that I too had given birth to a child. I used to wake up crying in the morning, cry during the day, and cry at night. Even the passersby, including the doctors, would tell me not to cry and to be strong, saying that God would help me. I felt comforted by their words because it's human nature, but for me, it was an extremely difficult time. Even when I was allowed to leave the hospital, my tears didn't stop. Getting into the car to go home was filled with tears. I was still in pain, and on top of that, I didn't have my child. My breasts hurt because of the milk. | | |
| All the postnatal care was good. For example, my sister would massage me with warm water every day. She would prepare meals for me every morning, and whenever I needed assistance, she was there to provide it. | | |
| I stayed with my sister because her place was close to the hospital, making it easier for me to return to the hospital if I felt unwell. That's why I chose to stay with her. | | |
| In urban areas, we may not use cultural practices as much as we do in rural areas. | | |
| It's true, sometimes hearing sorry can exacerbate the pain, especially if someone previously saw you with a baby bump and then suddenly notices the absence of the baby. It's better if they don't say sorry" if there's nothing else to say. Many people came to check on me later and asked about the baby bump, and I tried to respond briefly. I don't like delving into the reasons behind the situation, nor do I appreciate hearing sorry. I prefer it when people simply greet me without saying sorry. This situation can continue to bother me for a long time. | | |
| Thoughts were overwhelming. I'd engage in conversations, nodding along, but in reality, my mind was consumed with thoughts of my child. Someone would say something, I'd acknowledge it, but later I'd realize my mind was preoccupied with my own thoughts about my child. I'd forget what was said to me and push aside my own concerns. | | |
| There was a day when a doctor suggested getting someone to talk to me, perhaps a psychologist, but I refused. At that time, I was in genuine pain. They insisted on finding someone to help me psychologically, but I declined. I felt that I was okay; all I wanted was my child. I had no desire for such interventions at that moment because I couldn't see the benefit due to the emotional pain I was experiencing. | | |
| When I returned home, my sister suggested finding someone to help me, but I refused. I insisted that I was doing fine, although I would listen to her at home because my mind was calm there. However, being in the hospital environment was challenging. In our room, you would find people who had undergone surgery, some with their children moving around, doing exercises in the corridor. It was truly difficult. | | |
| Certainly, it would have been different if we were placed separately from those with children. Even though I would have been experiencing pain, it wouldn't have been at the same intensity as theirs. Imagine, we all went through the pain of childbirth, and they had their babies nursing, while I was alone there without a child. That was a significant challenge. | | |
| If there had been the capability, they could have been allocated a special area to stay where they would be alone, while parents with children were accommodated elsewhere. This would have been highly beneficial because, although the pain would still be present, it would have been somewhat alleviated. Such issues affect few, not many, but it would have had a positive effect to place them in an environment that offers privacy and peace. Even though the pain would have diminished, it's still very difficult to feel comfortable when you see others breastfeeding their babies while you're alone. | | |
| When relatives come to see me, I also cry, and their tears make me start crying again. | | |
| In my situation, the impact is felt when you realize I have suffered a loss and then you're told it costs a lot of money. | | |
| The child wasn't tested, and they were conducting numerous tests. After they tested me, they transfused me with yellow blood, of which I'm not sure what type it was. | | |
|  | |  |
|  | | |
| When I discovered that I was pregnant, I was truly happy. I was happy because it was something we had been trying to achieve for a long time. We had planned to expand our family, so upon discovering that I was pregnant, my partner and I were delighted. | | |
| My relatives were happy when I informed some of them, and my parents were also pleased. However, I did not inform the neighbors. They noticed my growing belly and started asking if I was pregnant, but I denied it, saying I was just full. I never told the neighbors that I was pregnant. | | |
| The midwife examined me and said, It seems like the baby has lost weight and has dropped significantly, even turning. Don't you feel any contractions? I told her, No, I don't feel any pain. Furthermore, before the thirty-sixth week, there was an error on my clinic card regarding the estimated delivery date. | | |
| The doctor asked me to describe how I was feeling. I told him that my belly had dropped, but I felt normal, and at that time, the baby was moving as usual. The doctor didn't examine me either and said that the baby might just be in an unusual position. He remarked that there was nothing surprising about the belly dropping, as it often happens around nine months, or the baby might simply be in an awkward position. This is normal. | | |
| The nurse insulted me very harshly. She said, You like wearing shoes, do you have any sense? What kind of manners are those? I told her, I'm sorry, I'll take them off after you finish examining me. She replied, No way, why are you wearing shoes in bed? I explained that I was unable to take them off. She said, If you knew you couldn't take them off, why did you wear them here? She insulted me a lot, but since I was already on the examination table, she continued to attend to me while insulting me. She examined me with annoyance because I had upset her. | | |
| The nurse asked me, Are you so-and-so? I replied, No, I am so-and-so. She said, If you’re not that person, why are you here? I told her, I'm sorry, I didn't hear properly when you called outside. Between my companion and me, one of us was supposed to come in. We were arguing, and my companion refused to enter, so I decided to come in. She insulted me and said, Please leave, leave, leave. You're coming in when it's not your turn. I said, I'm sorry, but could you please attend to me because I am next in line?" She said, Please leave here, go away. At that moment, she was scolding me while I was lying on my back, telling me to get up and leave. I had to lie on my side first to get up, but she kept scolding me. So, I went outside, and once outside, I felt very bad. | | |
| I went for the ultrasound, and the person conducting it seemed to be in the same irritated state as the first one. They conducted the scan angrily. That day, when I left there, I wondered why I was having such bad luck. They read the ultrasound results to me and gave me instructions. When I went home, I thought the ultrasound hadn't been done properly because they conducted it very roughly, to the point where my abdomen was hurting. That person was the one who had the ability to determine whether I would give birth that day or not, because it was around the thirty-seventh week, and it was a Saturday. If they had the willingness to attend to me that day, I could have even had surgery if necessary, and we both would have come out safely, because on that Friday, the baby was moving around a lot. | | |
| The next morning, I went to the hospital and was examined by a doctor who happened to be from the Chaga tribe. He told me, "What are you worried about? The baby is fine, active, and even causing a ruckus. The baby's heart rate is at 158 beats per minute, which is perfectly normal. Don't worry, just go back home and wait for the delivery day. | | |
| I went back home, but I didn't have peace of mind because even though the doctor said the baby was active, I couldn't feel the baby's movements. Even after drinking cold water and sweet things, I still couldn't feel any movement. | | |
| So, Friday evening, I had dinner and went to sleep, but I couldn't feel anything. Saturday was the same. I thought, No, let me go back to the hospital. My companion asked if I was feeling unwell, and I replied, No, I'm not sick, but the fact that I can't feel the baby moving in my belly is disturbing my peace. I want to go back to the hospital. | | |
| I went to the hospital again, but the results from Friday and Saturday were what confused me. I met with another doctor, a specialist in obstetrics and gynecology. She examined me and then advised me to undergo another ultrasound. I went for the ultrasound, and afterward, I asked about the gender of my baby. She replied, At this stage, your pregnancy is quite advanced, and determining the gender isn't clear. However, your baby is safe, yes, your baby is safe. When I kept asking more questions, she said, You know, these things have their own order. | | |
| The person who was supposed to interpret the ultrasound image for us was indeed a doctor. We went to the doctor for interpretation. However, as I looked at the doctor's face, I could clearly sense that something was not right. Whenever the doctor examined the image, their expressions seemed off, as if they had noticed something unusual. When I asked questions, the doctor would laugh as if everything was fine. Eventually, the doctor said, Some things are highly confidential. Go to your doctor he will the results; I've already sent them. | | |
| Participant 6 | |  |
| When I went to see the female specialist doctor who had examined me the first time, upon reaching there, when it was my turn, I went to see her for an explanation. She didn't explain much at first, but later she said, Now, we need to admit you. | | |
| I asked her, How can you admit me when I don't feel any pain? I was trying to differentiate between the initial contractions and the current ones. I feel like this level of pain isn't enough for admission. She replied, No, we have to admit you. I agreed, and when asked if I had come with someone, I said no, but if needed, someone close by could be called. | | |
| I asked about the ultrasound results and what they showed because when I came yesterday, I was told this, so I wanted to know the results of today's ultrasound. | | |
| She took a deep breath, and this was the question I had been anxiously waiting to ask. She said, In truth, your baby has passed away.  I asked, My baby has died? I was sitting on a chair, and it felt as if the chair lifted, and my bag fell. I felt drained of energy, but I managed to compose myself after a moment and sat back down. I asked the doctor, What do you mean? She replied, Your baby has died, he's passed away. Honestly, she didn't use any comforting language. She just kept repeating, Your baby has died, he's passed away. She pounded on the table, saying, "I'm telling you, your baby has died, he's passed away. | | |
| I asked her, How is it possible that yesterday I had an ultrasound and the doctor told me the baby was fine, with a heart rate of 158, but today you're telling me the baby has passed away? I don't understand. It would have been better if you had told me the baby's condition was critical and that we needed to do an operation to save his life; I would have understood that. | | |
| She told me repeatedly that the baby had passed away and was no longer in this world. She said it about four times, and another colleague of hers repeated the same thing: Your baby has passed away, he's no longer in this world. There was no room for negotiation, even if you suggested an operation, it didn't mean you would save his life. I was truly hurt because she was giving me answers that were painful. I left without saying goodbye. I was tired of hearing those words, he's dead, he's dead, so I decided to leave. I didn't even care about being admitted. I just picked up my bag and left the hospital. | | |
| I left and felt like I couldn't go home properly, so I found a place to sit and called my companion. | | |
| I was confused; I couldn't believe the information because just yesterday, around eight o'clock, I had been examined and told that the baby was fine. Then, the next morning, I was told the baby had passed away. | | |
| I called my companion before leaving to go back home and told them about the information I received at the hospital, that I was told to be admitted and also that the baby had passed away in the womb. They couldn't believe it and said, How is it possible for the baby to pass away in the womb? | | |
| Yesterday you told me the baby was fine, and you were attending clinics as usual. Because they couldn't see anything wrong during the examinations, this doesn't seem possible. We really needed this baby; we were eagerly waiting for him. | | |
| Even when I explained, they said they didn't believe it anymore. They told me to wait for them, that they were coming. After finishing work, they arrived, and we went to another hospital first to confirm everything before proceeding with any further decisions because we were unsure. | | |
| When they conducted the ultrasound, just like usual, they seemed to see something unexpected. At first, they were cheerful, but then, when I started asking questions, they pretended not to understand. They said, "You know, this ultrasound procedure requires a certain level of expertise. It's like when you enter a new house, you need to first understand the environment before you start explaining. They told me to calm down, saying they were still in the process of understanding. They kept focusing on the screen but appeared uneasy. When I asked, they said, I'm still searching for something to explain to you. Please, let me concentrate, don't disturb me. | | |
| Participant 7 | | |
| Truly, that brother, I don't know if it's the expertise they are taught or his own tactic, but I couldn't understand. He just left me lying there and walked away. He went and told the person who brought me to come and get me. Honestly, he avoided explaining to me. He said he would bring something, maybe if there was something missing. I continued lying there; he didn't even wipe off the gel or the ultrasound gel. He said he would bring something, then someone else came and wiped off the tissues. | | |
| When I drank my tea, I expected him to move, but he didn't. I started crying right there at home because his lack of movement felt unusual. The fact that he wasn't moving, something that was usually routine, deeply saddened me. | | |
| The pain started at home after feeling that the baby wasn't moving as usual. I already knew in my heart that something was wrong even before the first ultrasound when they said the baby had passed away. When I saw the same thing in the second ultrasound, it confirmed what I already suspected. | | |
| He received me well initially, but later, during the ultrasound session, his demeanor changed. His facial expressions altered as he zoomed in on the images, then quickly returned to normal. It was as if he was avoiding eye contact and abruptly stopped the conversation we were having. That's when I realized he had noticed something unusual. | | |
| Their response was similar to that of the other hospital, and the tests showed that the baby had passed away three days before today. | | |
| I was deeply hurt because hearing that the baby had passed away affected me more than five times. When they told me to sleep and wait for contractions so they could figure out how to assist me, it added to my pain because I felt I couldn't even sleep alone. | | |
| I was experiencing pain, to the point where I wished someone would be there to talk to me until dawn. | | |
| There's a difference. Starting from that Friday when I began to feel different, the sensation with the baby who had passed away felt heavier, like I was carrying a heavy ball. With a baby who's alive, their movements aren't as heavy. So, when I slept from Thursday to Friday morning, the baby seemed to be pressing heavily on one side, to the point where my legs felt numb, unlike previous days. | | |
| The pain became more intense, perhaps because I felt differently; it's different when you're struggling to connect with your child. | | |
| I was worried because of the negative responses my husband and that lady gave, despite the initial affection they showed. She even said, Tonight, we'll all be on the journey of labor pain together, so her responses made me anxious. However, I thank God she wasn't there when I gave birth because if he were, I would have been even more afraid. | | |
| They took him and cut the umbilical cord. After that, they told me, "This is your baby." I really wanted to hold him, but when I did, he was so stiff, with his eyes closed. I thought maybe if I shook him a little, he might wake up and cry. Even when I held him, he was still warm. I couldn't believe he was gone; I thought he would cry later. | | |
| I saw his face, I saw his body, and even today, his image is still vivid in my mind. I wished I could do anything to make him wake up. I kept thinking, and when I was there, I couldn't believe he had really passed away. Then they took him, and I told his father maybe he wanted to see him, so they called his father and took him to see him. | | |
| They took him to the mortuary, and I was worried that if they injected him with a needle and put him in the fridge, he would die completely. But later, I was told that they don't do that to young children. I thought to myself, If he gets hungry, he'll cry. | | |
| They took me there, to the postnatal ward. Indeed, the postnatal ward is for those who have given birth and those who have lost their wards, and they must be separate. | | |
| Since that baby was my second, I don't know if it was loneliness, but I felt an unusual coldness. I couldn't even hold onto my cup of tea; my body was covered in goosebumps. It felt like I had experienced loneliness; I was separated from my child. You know, being with a creature for nine months, I had already grown accustomed to it. | | |
| It's like he was still in my body; I would talk to him even though I couldn't see him. I would say, Grow, my child, I love you, and I'm waiting for the day when you grow up and I can see you. When I said that, he would move, so I believed he was alive. I prayed to God for him and poured blessings on him, wishing for the day I could hold him and talk to him face to face. | | |
| I wished I could stay with him, talk to him, and even give him a kiss, even though he was small. But they ended up just showing him to me and then taking him away. | | |
| I wished they could have given me even just five minutes, but they didn't give me any time at all. I was lying there, and they hadn't even removed the placenta. They just showed him to me from the side and said, This is your baby. So, I couldn't even sit up. They didn't even put him in my hands; they just held him by his head and fingers. I wished I could have held him even for a moment, maybe touch his face, but they took him away too quickly. | | |
| Participant 8 | |  |
| When they took him away, I didn't see him again or hold him again, and they buried him while I was still in the hospital. Later, I was told that the baby had been preserved there. | | |
| When I desired to even touch him on the face, they took him away to preserve him. I was still there when I later learned that my child had been preserved there. Why was he preserved? He was preserved because he was a fully developed human being, not just a miscarried fetus due to bleeding or other complications. He was fully formed, so he couldn't be discarded or left at the hospital. Leaving him there would have caused more emotional distress. We had prepared to care for the baby as we had saved up and made plans. Therefore, preserving him was better, even though it seemed like reopening old wounds. But it's better knowing that my child is in this place. When they asked me if I agreed to have the baby taken away, I said yes because I didn't know when I would leave the hospital, and I was afraid he might deteriorate while waiting for my discharge. So they took him to be preserved, and my husband was also present. | | |
| According to our cultural norms, which I wasn't very familiar with because I was born in the city, when I asked, I was told there are two ways for a new born baby: if the house doesn't have cement, they dig a hole inside the house and bury the baby, but it doesn't cause any bad luck or invite spirits because I asked and was told so. Also, the second way is that if the baby is not buried inside the house, they dig a hole either near the door or near the wall, and that's where the baby is buried, not far from the house, like a baby's cradle. So, we were given those two options. | | |
| Yes, we buried the baby at home. | | |
| So, when people came to see me there, I felt bad. They came to offer me condolences, but I wished they had found me busy breastfeeding the baby or receiving the child. They would come and look around, and I pretended I was okay, talking to them. But when they left, I felt a deep chill when I was alone. Meanwhile, my friends were interacting with their children, and I really wished I could join them.Even though they weren't treating me badly, I longed to be with them and teach them, too. | | |
| If the government could allocate separate rooms for parents who have given birth to babies who have died and those who are alive, it would be better. I think meeting five or ten people in one room, first of all, will offer mutual support even if you don't speak, you will feel like, Okay, I've lost, but so has the other person. But if you are alone in a room, you will feel like the whole hospital is mourning only your loss. | | |
| It's better if they stay in their own rooms. There, they can comfort each other, cry together, pray together, and console one another. Eventually, they will all see that these are normal things to go through. | | |
| It's hard to forget the experience during the 37th week, especially with the mistreatment you endured. It seems they failed to provide you with proper care, despite having the capability to save the baby's life. It's understandable that such memories would make you reluctant to return to that hospital, even if you had the means to do so. | | |
| And what happened after your child was buried? Initially, while still at the hospital, I was crying, sleeping, and waking up to the reality of the situation. My husband came to pick me up from the hospital, and returned home. During the journey, he asked what I wanted to console me, but i remained silent. | | |
| When I arrived home, I found my relatives there, including my siblings and his. Seeing them there really heightened my pain. I also encountered my eldest daughter, the child from our house help. It was painful because when a mother goes to the hospital pregnant, everyone expects her to return with a baby. But in my case, I returned empty-handed. Now that I was back, I was supposed to explain, but they were comforting me, and I kept telling them I hadn't brought any gifts. | | |
| The neighbors came to know later because in our community, the death of a young child is not usually announced as a funeral. We don't make announcements. They came to know by asking, realizing that I had given birth. I told them it was true, but it wasn't meant to be. Once one person knew, the others would find out too as they would spread the news. | | |
| The care was good; my mother went to great lengths to provide me with maternal care, even though she already knew the baby had passed away. Personally, after losing the baby, I felt worthless, although they still saw value in me. I worried that my husband would be angry or see me as negligent because he had incurred expenses for the pregnancy, but they took care of me well. I felt ashamed, feeling like I hadn't done any work even though I was eating well. I wondered how it would have been if they hadn't cared about me; I wished I could prove myself. | | |
| When you're planning to add a child, sometimes even if you, as a woman, don't want to, but the man does, I wondered how it would be if my partner demanded it and I couldn't fulfill it. Would he accept me, or would he seek someone else? Would he see me as unlucky and mistreat me? But he never changed; he encouraged me and took care of me well. If it weren't for others caring about me, personally, I wouldn't have been able to care for myself. There were times I felt worthless, like I hadn't done any work for nine months, just bothering people, and in the end, I lost the baby. | | |
| It's true that it's God's plan, but I feel like I've made a mistake too. Maybe I should have insisted that the doctor do something if I had known. I wish I had gone to the labor ward myself, but how would I have known if it would work? Next time, if I get pregnant again, even if I have just two weeks left, I'll go to the hospital for delivery, not just any hospital but a big one. We'll be paying the doctor a lot of money so they take care of us. These other hospitals, where you're mistreated and not given proper care, they fail to understand your challenges. | | |
| During that week when I was insulted so much, he could have known if the ultrasound really failed to show. | | |
| Truly, the government should pay close attention to those studying in the healthcare sector because the way they insult pregnant women is not right. Negative remarks towards pregnant women lead them to fail to express how they feel. They should be compassionate and provide proper care without insulting us. | | |
| Another thing I learned is that when these challenges arise, they haven't just happened to you alone. They've happened to others as well. You learn that there are people who have experienced similar difficulties but still went on to have children. So, you realize that it's not just about you; others have gone through it and had their reproductive issues resolved. | | |
| Yes, it would be helpful. However, opening up to someone you're not familiar with can be a bit challenging. You might find that they're just listeners of the conversation and not active participants. It's not like a WhatsApp group; it's a group at the hospital where you gather to share your medical history. | | |
| Indeed, having a dedicated ward where parents who have lost their babies can gather and support each other would be beneficial. Additionally, having a psychologist available in the bereavement ward to provide emotional support, listen, and offer guidance to grieving parents could have a significant impact. This would provide comfort and allow parents the opportunity to express their feelings openly. Establishing such support mechanisms can greatly contribute to the healing process for parents who have experienced the loss of their babies. | | |
| In the room where other parents have successfully delivered their babies, doctors would come for rounds and attend to them, observing their infants. However, in my case, they would pass by without attending to me. Even if I was lying down and appeared fine, I couldn't help but think that perhaps the reason they were skipping me was because I had experienced a loss. | | |
| But when you are all in one room, even if the doctors don't come frequently, they don't miss anyone. When it's time for medication or encouragement, they attend to everyone. | | |
| Psychologists help to build a mentality in patients so they do not feel isolated or responsible for their condition. | | |
| Before being allowed to care for a mother at home, caregivers should receive education on how to treat her well. Otherwise, she may feel neglected. Without proper education, even fathers, if aggressive, may verbally abuse a woman, accusing her of causing the child's death if it dies in the womb or had challenges. Consequently, family members could lead to suicidal thoughts or the mother leaving home. | | |
| I wish I knew why my child is facing these challenges, but right now, I just feel overwhelmed. I wish I knew the source. | | |
|  | |  |
| Participant 9 | | |
| he took that positively and said this time it can't be like what happened before. he said it will be safe. When I got pregnant, I didn't want anyone to see me, so I try to stay indoors a lot. Most of the time, I stay indoors. I'm afraid that at some point, it might happen again. I don't want people to judge me. | | |
| As the days went by, I noticed that the baby wasn't moving, even though until then, everything had been going well due to the medication. It just happened that the baby wasn't moving. | | |
| The movements decreased, so I contacted my doctor, and he told me to go to the hospital. I went to the hospital, and they checked with an ultrasound, the baby was fine, but it appeared that the amniotic fluid had reduced. After the amniotic fluid decreased, I continued bed rest while still receiving treatment for my blood pressure (I was at home). After two days/the next day, I felt the movements decreasing further, so the following day, I couldn't feel the baby moving at all. Therefore, I went back to the hospital for another ultrasound check, only to find out that the baby had passed away. | | |
| I was extremely scared because it was something unexpected. I felt like I had surpassed the earlier risks that usually occur. So, seeing the baby not moving again at this point increased my fear even more. It felt like the fear intensified. | | |
| How was it during the ultrasound until they told you…? It was because the first time, I was just told that the amniotic fluid had decreased, but the baby was alive. While I was trying to console myself, I still had a great fear. The fear was even greater because I didn't have any hope that the baby was alive; it was fifty-fifty. It was smaller than what I had imagined in terms of the baby being alive, so I was very afraid because I didn't want to receive negative news. I hoped to hear that the baby was alive, but the results turned out differently. | | |
| I was given the results immediately after they finished examining me. Because I had previous experiences that had already happened, the doctor seemed to also be hesitant in giving me those results. But later, he gave me what he had to say, mentioning that I had contacted the doctor. I told him yes, I had contacted. He then asked me about how many pregnancies I've had and how many children I have. So, based on those questions he asked, I immediately knew that the baby had already passed away. So, after everything was done, he gave me the results, saying I should take them to my doctor for interpretation. But later, considering his previous situations and since I had already told him, he decided to interpret the results for me. So, even I myself, before taking them to the doctor, I had to open them to see what was in the doctor's report. When I read it, I understood, but I had to read it again. And after taking them to my doctor, they had to redo the tests, but the results showed the same thing. | | |
| Aaah, I thank God, I had just gotten the courage and strength. I got it because the pressure was already high, so I said to myself, here, if I continue to bring more fear, I might end up causing myself even more problems. Truly, God gave me strength too. So, I became brave, and I accepted it. | | |
| Yes, I had my husband with me in all the situations I was going through, I was with him. He agreed with it, he accepted it. So, they had to start staying with me and bring me back to health. | | |
| From the moment on the ultrasound where I was told the baby had passed away until before I was given the epidural injection... I was praying to God a lot. I found myself stuck in prayer so that the baby would come out safely. You know, delivering a baby who has already passed away, it was painful, but I was praying to God a lot. I just wanted to come out of it safely. | | |
| I was afraid, I felt the pain of losing the baby, but I was also looking at myself so I wouldn't get lost in it too. | | |
| I wish it would happen quickly, unlike how slowly it's going, because the pain I'm experiencing in labor pains is excruciating. It's not that I'm hurting because I've already lost, I'm hurting from the pains of labor now. So, I wish it would happen quickly so that I can heal too. | | |
| Aaah, there was a doctor, there were nurses, and there were also nurse assistants, and a night doctor, I think they were there. They were in a state of watching over me, because of the condition I was in. Honestly, they provided me with very thorough care. In the hospital, I received extensive care and treatment, apart from the help from God. But they tried to do what they could to help me. | | |
| When they received me, they showed me, saying, "Your baby is this one here." It was this one, of this gender. Then, they told me that they would take him to the mortuary until you arrange to come and pick him up. | | |
| I didn't want to, because I knew it would only bring me trouble if I continued to look at him. But later, I didn't regret it either, it just brought me pain during that time. However, if I had been asked, I wouldn't have accepted to see him. | | |
| Honestly, they were in a service-oriented state, they were good in service. They were very close to me, and I was also given counseling. It helped me a lot, and a doctor also came to counsel me. So, I accepted. | | |
| All of them, doctors and nurses, they all used to come regularly. They didn't leave me alone for long, every now and then, they had to come and check on me. | | |
| I encountered difficulties after returning home. First, I found myself alone with my sister; everyone else had gone about their activities. Being at home in that state tormented me greatly. I was constantly wondering, asking myself, why? why? Why? This self-questioning caused me trouble at home (cries). I'm sorry. So, being at home took a toll on me for quite some time, it took time. This led to increased pressure because of the questions I asked myself. It caused me trouble for almost a whole month before I started feeling better. Then, after being taken for counseling, I spent some time, about a month or so, before I was able to move on with my life. | | |
| Aaah, my husband had to take leave, so he was very close to me. Most of the time, he made efforts to comfort me, and that's how it was. He had to take leave so that we could go to the hospital together, and even the counselor himself was comforting me. Until I felt better and realized I had to stand up again. | | |
| God has given me life, and now I need to start a new chapter. So, I had to stand up and start moving. Even moving around was helping me. I go to do my business, I go to church. | | |
| Because I have never buried anyone myself, I have never been taken to do so because whenever such things happen, I am usually in the hospital. So, relatives, gravediggers, they just go and bury as usual. | | |
| And where are they buried?.... At home, we have a farm, so they usually go and bury them in our fields." | | |
| They took me to a ward where I was alone. Honestly, being alone in the ward also helped me. I think if I had been placed in a ward with other mothers and their babies, I might have experienced more pain. Being taken there, I also requested to stay alone, so I wouldn't hear or see other mothers breastfeeding or caring for their babies, which would have caused me more pain. So, I just felt normal there, continuing to pray to God to work things out differently next time. | | |
| I requested, and when I was in the ward, I was assigned a relative to stay with me. I had a relative with me at that time, and it helped me a lot. | | |
| I feel burdened because I wonder what the problem is. If I'm receiving good treatment, then what is the issue? Another thing that bothers me is that I see others with high blood pressure but still give birth to healthy babies. So, I wonder why my babies pass away. This really troubles me a lot. | | |
| If she herself has involved the community, then the community should support her in any way, but it should be in a receptive manner. However, upon returning, if she didn't receive any support, I felt it was better to quit the job and do other things. | | |
| If they're not supporting her spiritually, then she might consider seeking support elsewhere or finding solace in her own spiritual practices. | | |
| Counseling should indeed be emphasized greatly in hospitals. They should sit down with the person and provide extensive counseling, especially if possible, not immediately after seeing the baby as if the baby has been delivered and then allowing them to leave home just because they're physically healthy. Psychologically, the person may not be well, so even if they're allowed to return home, there should be a system in place where they seek counseling regularly, perhaps every week, considering the experience they've been through. | | |
| For someone who has a history of losing children, I think it might help if, whenever they become pregnant again, they stay in the hospital until they give birth. | | |
|  | |  |
|  | |  |
| Participant 10 | | |
| I was admitted to the hospital, and I was maintaining my blood pressure, but later I heard that the baby was not moving in the womb. | | |
| With a small pregnancy, I suffer a lot; I feel sick and even vomit. Whenever I go to the hospital, the issue is always about blood pressure. | | |
| Personally, I'm not in a happy state because the human mind tends to feel that even what I'm carrying, I might lose. So, you feel that... I feel like I might even lose what I'm carrying. | | |
| So, what state are you in when you feel that way? Are you normal or how do you feel? ... I'm normal, but I suffer a lot, I mean I suffer a lot. | | |
| The main thing is I struggle to breathe; the pressure bothers me a lot to the extent that once I conceive, the pressure becomes very high. | | |
| Once I reach around three months, the pressure starts to become high. | | |
| So, when I got this pregnancy, I started going to the clinic. When I arrived at the clinic, they immediately checked my blood pressure, and I was given medication which I used. However, when the pregnancy reached six months, the pressure became high, and they transferred me from the first hospital to the second hospital. At the second hospital, I underwent tests and was admitted for about a week. | | |
| Personally, for this last pregnancy, because in all my previous pregnancies, I myself didn't feel that I was very caring or cautious about the pressure. But for this last pregnancy, I knew I would get it because I took a lot of care about the pressure from the moment I conceived. Pressure bothers me a lot once I conceive, so I took care of it from the early stages of pregnancy, taking the pressure medication properly and caring a lot about how my pressure was. For this pregnancy, I knew I would get it. | | |
| I didn't feel well because I already had hope, but now I felt like I was failing even though I had already placed hope. | | |
| I was transferred to the third hospital and stayed there for a month and a half until the baby came and died in the womb. | | |
| I used to hear him play, mmmmh, I used to hear him play, but at some point, I started having difficulty hearing him because suddenly the weight of the pregnancy increased, and the belly became bigger, as if it was filled with gas. So, it bothered me even to listen to the baby's heartbeat on my side. Even when we sat with others, while they could measure, it was difficult for them to measure mine. | | |
| Yeah, I just knew because I used to know every day during this time the baby would play, and they would come and measure. But suddenly, for a whole day, I didn't see him playing. | | |
| Mmmh… They measured me, when they measured me on that first day, they couldn't give me answers, and they couldn't give me responses on the first day. They came back and gave me answers on the second day, but even so, when I asked them myself. | | |
| They tested me using ultrasound because with the regular pregnancy monitoring equipment, they couldn't hear anything. | | |
| On the first day, they didn't come, but on the second day when they came to my room, I explained to them how I was feeling. The doctor asked me if I had received any feedback from the previous day's test, and I said I hadn't. The doctor then asked who the person I knew was, and I said yes. The person was called, and they were asked why they didn't bring back feedback from the previous day. They replied that they couldn't bring back feedback, but they administered an injection to prevent infection because her condition was critical due to her very high blood pressure. | | |
| They said that due to my high blood pressure, it caused a problem with blood flow to the baby, resulting in the baby passing away in the womb. | | |
| I was given the feedback with the doctor who initially examined me, along with a whole team of doctors, students, and fellow patients who were nearby listening. | | |
| I felt very, very bad because it was something I was expecting, something I knew that there was no room for failure here because up to this point, in this hospital, where I had reached the end, I had so much faith that I wouldn't fail because of the excellent care I was receiving. I couldn't believe it because even the doctors themselves were shocked because they couldn't believe it. They had already received and understood the problem, and even they were hurt by how to deliver that news. | | |
| I knew they were hurt because when you say someone is hurt, even when delivering that news, you can tell by their expression. You know they are hurting because even within that team, one of them who was conducting the tests today, the next day when someone else came to deliver the news, that person was visibly saddened, like, Why here? We fought so hard here; we were expecting to have a baby. Mmmh. | | |
| In the human mind, if I felt that way because usually, when you have an ultrasound and results are available immediately, and sometimes you get the results right away. Hmm, but on that day, they didn't give me the results. | | |
| So they left and didn't come back again. I didn't see them anymore, so I started to feel worried. | | |
| For me, it was really tough; I was in a very bad state. Even though I found comfort in my companions, my siblings who came to see me, I told them about my condition, saying, Right now, this is the situation; I'm just waiting for the moment to deliver the baby. | | |
| During that time, it was just agony that I went through. I endured immense pain because of those medications. I feel like the dosage was increased, not just because I felt it, but because he himself admitted to it. | | |
| The doctor provided me with good care, and I received medication promptly. My relatives brought me food on time and they were attentive to my needs throughout. | | |
| My feelings during labor were of intense pain because I knew that I would deliver but not have a baby. Personally, I didn't feel well because it's better for someone who is going to deliver to know that they will have a baby afterward, but I already knew that I would go through the pain without holding the baby because I already knew that the baby had passed away in the womb. So, I wasn't in a good human state because every other woman was struggling and crying when her baby came out, and she was happy, but I wasn't feeling that happiness in my heart. However, I was there, trying to accept the pain just to deliver the baby from the womb because that's the way out. | | |
| He received my baby, then told me, Here is your baby. But, you know, the baby had already passed away. I told him that I knew. He then asked me if I wanted to see. I said yes, show me, and he did. | | |
| He held the baby like this and showed me while I was lying down. Right after he showed me, he took the placenta and cleaned me up. | | |
| I wished the baby had been alive. I wished I had given birth normally, with the baby alive. But there was nothing I could do because the baby had already passed away. | | |
| How was it when he showed you? Was it appropriate or not?... It was because I needed it myself. | | |
| No, I just looked at him because with the pain I had in my heart, I couldn't bear to stay with him any longer. | | |
| I went back to the ward. | | |
| The baby had already been taken to the nursery, where they placed him in one of those baby cribs. After that, they took him to the mortuary. I went back to the ward and continued with my blood pressure and magnesium drip. On the second or third day, when the medication finished, I was discharged. | | |
| How does it happen?... They take him, recite prayers over him, wrap him in a white shroud, and bury him with the guidance of an Islamic leader. | | |
| My friends who were also admitted came to visit me, so I felt hopeful because even in the ward where I was admitted, there were others facing similar challenges like mine. We talked and they comforted me. | | |
| It helped me because deep down, I realized I wasn't alone. Knowing that others were going through similar challenges like mine comforted me. | | |
| In our ward where we were all admitted, we were all in similar situations. One had undergone an operation but didn't have a child, another had undergone an operation but couldn't conceive. Someone else might say, "I've given birth three times but have lost each one." We were many, and seeing others going through similar struggles brought comfort. It made me feel like I wasn't alone. | | |
| While I was in the hospital, to be honest, my relatives took very good care of me. There was never a moment where I missed medication or felt lacking in any way. Perhaps I missed a dose due to meal times overlapping, but no, there was nothing missing. They were there for me all the time when I was unwell. I was lifted, cleaned, and attended to for all my needs by my relatives. | | |
| At home, personally, the challenges are not comparable to those in the hospital because, in the hospital, when you feel like resting, you can rest. But at home, there are noises, and you may need to do things, but nothing was lacking. I received excellent care. I was bathed and attended to just like a patient. | | |
| I received care for about one and a half to two months. | | |
| No, there were no cultural rituals. I was just recited prayers, and I called a cleric who came and recited prayers for me. | | |
| Personally, there was nothing specific that I wished for to be done for me that wasn't done because I knew what my problem was, and I was taking care of it. I don't blame anyone, not the doctors nor myself. | | |
| What I really wanted was for the doctors to be caring towards the patients. | | |
| Personally, during those first few days, I felt very vulnerable, to the extent that even going outside seemed daunting. | | |
| I might go outside and see my friend who has a baby while I don't. There's this feeling deep inside that hurts because it's a long journey. But the most comforting thing is the support from neighbours and close friends who came to visit and comfort me since I got out of the hospital. I'm grateful for friends, relatives, and family; they give me comfort. I don't lose hope, and I stay close to the doctors | | |
|  | | |
| Participant 11 | | |
| I accepted it because I needed it, I didn't have a problem with it. So I just accepted it wholeheartedly. And my partner also accepted it wholehearted | | |
| It's like I have this tendency that whenever I'm faced with such situations, I just collapse (laughter) you know, you become thin, very thin. When you go to check, you find, well, I just thank God because it was my desire that God would grant me a last child, so it turned out that way. | | |
| Ah, when they found out I had high blood pressure, they immediately started me on initial treatment. It was there that they began my care, and then they referred me to the hospital. | | |
| They referred me, and I was treated there for about two weeks. When I left, I was doing well, so I continued going to the clinic every week | | |
| I was hit by a cold, and when the intense cold hit me, I stayed, and it passed by morning. When I woke up, I went to the hospital. Even when I went to the hospital, the baby's movements weren't good. I went to the hospital until the laboratory, ultrasound, then the doctor gave me the results that the baby had died in the womb. | | |
| Ah, before I went to the hospital, before I went, the baby's movements weren't good. | | |
| You see, you find your heartbeat, he plays for a minute, then for a long time, then plays again. So even when the doctor examined me before I even went to the lab, he told me, Why is your heartbeat so low? I told him, Even I don't understand what's happening, this movement is not normal. | | |
| You find he plays once, alright, then he stays still for a long time, which is not his usual behaviour. | | |
| The ultrasound didn't reveal anything, but I could hear him laughing. | | |
| The doctor who examined me during the ultrasound was surprised when he noticed something, so he called his colleague. When his colleague came, they both agreed, saying, Yes, yes, yes, I can hear them that the baby has died in the womb. | | |
| I was truly shocked, but I didn't want to show it. | | |
| You see it, I didn't want to show it. Then I went back to the doctor to return the results. I met the doctor, and he told me, Well, the tests say this. I didn't have any choice as a parent; I felt saddened by that, I cried, then I got up and continued with another step. The doctor then wrote something for me; he said, Well, in this situation, you have two options in the ward. Either you give birth naturally or through surgery. How do you usually give birth? I told him, I usually give birth naturally. So, he wrote something on a piece of paper for me to take to the ward. But I went back home first because when I went there, I didn't have money, I didn't have clothes. I had to return home first to gather myself. I returned home, collected my belongings, took some money, and then went back to the hospital. When I arrived in the evening, I was admitted. | | |
| aah, you know, everyone expects to find what they're looking for. We don't expect to miss out; most of the time, we expect to get what we're looking for. So, when you've already found that thing, and then you lose it, as a human, you naturally feel weakened. | | |
| Yeah, in those moments, it's like... (laughs) I mean, it's hard to find the right words sometimes, isn't it? (laughs) It was tough, but you had to keep it light, you know? Given the circumstances, you've carried something for so long, hoping to find it, and then you don't. When a parent's hopes of getting what they've been expecting vanish, you see, it's like your spirit just goes a bit numb. | | |
| He asked me if I remembered that moment when I told him that the baby's heart rate was low, and I confirmed it. Then he brought up the baby's movements not being right, and I confirmed that too. He asked, So, what's the situation now? I replied, "He's not moving at all. That's when he told me, The tests show that the baby has passed away in the womb." I acknowledged that I understood what he had just told me. | | |
| I walked home because I didn't have any money with me, so I went home, took some money and clothes, and left with my neighbour. We went back to the hospital. | | |
| I told my neighbour that the issue was still at the hospital, and we had to return. There was no other option. So, I took my money and clothes and left for the hospital again. | | |
| When I arrived at the hospital, I was given a bed. Around 10:00 PM, they administered the first dose of pain medication, then another dose at around midnight, and another in the early morning hours. | | |
| it is hard, because you know all labor pain is for nothing, I was in labor only to save my life | | |
| They took good care of me when I needed assistance. They were there for me until I gave birth that afternoon on the tenth. I'm grateful for their services; I was truly well taken care of. As for others, I'm not sure how they were attended to. | | |
| When you're in the hospital, you know your relatives will come, bring you tea, and someone will bring you porridge, at least to comfort you. They won't leave you alone. | | |
| So, after giving birth, my relatives came in the evening to take their child and buried it. I stayed there for about two days before I was discharged. | | |
| The child was placed near me, not far away. While lying down, they moved the child closer to me and said, Look, the doctor said its candida. See, this is your child. I responded, Oh, which gender? They said, It’s a boy, yours. I replied, Okay, thank you. After that, they covered him properly and took him to the mortuary. | | |
| That's true, acknowledging the role of a higher power can provide solace and strength during difficult times. It's important to recognize that some things are beyond our control and to find comfort in faith and trust in a higher purpose. | | |
| during the burial process, my husband took charge of the arrangements instead of me, as I was still in the hospital recovering from childbirth. | | |
| They buried the child as usual. There aren't many elaborate rituals involved in burying a child like that. It's more about dressing him in clothes and burying him | | |
| Just one day... it was the same day, I mean, when they buried that child. For us personally, they dug a grave as usual and placed the body in it. Eventually, when I recovered, I went to see this grave here. | | |
| So, when you meet your peers there, you find that they have also missed [their babies], and some of them are even breastfeeding. | | |
| Ah, then you realize that it’s not just you, but a challenge for many others as well. It's not only you experiencing this, so it's important to just thank God. | | |
| Because we were there with those whose children go there to breastfeed, those whose children were still young. So, during the breastfeeding time, they would go to breastfeed. Ah, as a human being, as a mother, it must be painful that you too could be breastfeeding there. But at the end of the day, you have to be grateful. | | |
| Comfort How so?... A nurse might come and ask how you are doing, offering you comfort, saying that you are not alone in this world. The most important thing is to thank God. That’s how it was. | | |
| You know, when someone comes to you and says, I'm sorry, that’s life, you see, everyone has their own problems,' deep down, what do you feel? You are hurting. Even though you accept it, it still hurts. | | |
| Ah, the only thing I wanted was a child, but now I have none. There is nothing else. | | |
| Yes, you must feel pain because this sympathy is given to me because I lost a child. But if I had a child, I would have been congratulated. | | |
| That comfort someone gives you, yes it hurts, but it helps, because it takes you away from those thoughts and brings you to other thoughts, like there is a tomorrow and you will get it. You see, don't lose heart if you missed today, but there is a day tomorrow you will get it. That’s the joy of having people close to you, comforting you; that’s the joy. | | |
| When I was discharged from the hospital, I stayed at home and was taken care of as a parent (mmmh). | | |
| I mean, regarding food, you know, there's that postpartum diet. So, I ate as usual and took my baths as usual, you know, like the usual postpartum foods. | | |
| I was taken care of for a whole month because my sister in-law also left home and stayed for a whole month before she left. | | |
| It helped me because her presence for the whole month meant I regained strength, you see, because she made sure I was okay before she left. | | |
| You find that they didn't leave you alone for long, you know, in situations like this if you're left alone for long periods, that's when you might feel like you're going back to where you started. | | |
| Meaning mentally, yes, you start recalling that image when you're alone, the one you had during labor (laughs). | | |
| You see, so when you stay alone for a long time, that image starts to bother you. So that image kept coming back... oh, quite often. | | |
| It's like when you've had a child and seen them, but then you lose them. You see, being alone like that gives you trouble. | | |
| In that case, it's better to have seen him than not to have seen him, because otherwise, I would have asked questions about where they took him, so it's better to have the painful truth, yeah. | | |
| Yeah, it's better to have seen him, even if it hurts, yeah. It's better to have the truth and know where he's gone than not seeing him and not knowing where he's gone, you see. | | |
| Yeah, exactly. When I saw him, it was enough, but now that feeling, you can't forget it for these recent days. It will fade, but not easily, slowly, slowly. | | |
| It's like that, when I was alone, my goodness, I saw my child and then he left, like that. Oh, as a human, I was just crying there, you cry and then you sleep, and that's it, no other issue. | | |
| In society, they tell you that nowadays childbirth is problematic; you might have a good birth and then later have a bad one, that's how it is. So, when everyone encounters a problem, each person has their perspective, each person has their thoughts. So, for someone with a problem, you have to have a firm stance, you see. Someone else might tell you it's not normal. | | |
| That's it. You find someone telling you it's not normal, how did the child come to harm you see, you're always just cold, let the child pass away you see. | | |
| Ah, now there you find that if you have a small brain, then someone will try to persuade you to go to the other side. | | |
| On the other side, meaning if you don't have a strong stance, they may persuade you to go to traditional healers. However, in reality, if you're the one with the problem, you need to have a stance. You must accept the outcome that this has happened because you're the one who faced the issue, not the one telling you what to do. You know the cause, and you decide the outcome. Another person advising you might give you different information that's not reliable. The key thing is to follow what doctors say and what is necessary.Top of FormBottom of Form | | |
| I was just there, spending time with two or three people until my forty days passed. I kept myself occupied by going to church and returning, and life continued. Now I find myself settled in this way. For instance, a problem like that truly exists a hundred times over, because it has already happened. But delivering news to a patient, you know, when a child dies in the womb, you may talk about it casually, but for the person experiencing the problem, carrying that burden is difficult if they aren't emotionally prepared. In my opinion, even delivering information needs to be carefully considered. Often, patients are bombarded with information, using language or phrases that can confuse them. In the end, you may find them in the ward shocked later on. It's better to have counselled them beforehand rather than just telling them abruptly that their child has died in the womb. That's a bit tough. | | |
| They should first provide counselling and advise, you know, gently prepare them mentally. You see, it's easy to give information, but as humans, it's hard to receive it. Giving information is one thing, but receiving it is another. Not everyone has a strong heart; we are different. Some have small hearts. If that information had been given to someone else with a weaker heart like mine, they might not have coped even at home. | | |
| Another thing I think about is even in the example of the ward, a hundred times over they stayed alone, those people. | | |
| Because you know that feeling, let's go breastfeed, or you find someone already breastfeeding and you've missed out, it's a challenge mentally, in your mind. Okay, my friend is breastfeeding and here I am, my child has already passed away. At least when they are there by themselves, they encourage each other. | | |
| It's exactly like that, I was told, my friend, let me tell you, the child died in the womb, you see, it comes just like that, as it is. Don't you find that difficult, my friend? | | |
| Ah, this really affected me at first because I had never experienced it before, you see. I never thought it would happen to me (laughs a little). Really, the thoughts wouldn't go away, I just felt foolish. But at the end of the day, you have to thank God and let other aspects of life continue. The main thing is to thank God. When you find yourself in that situation, you turn to prayer and God grants you strength to continue with life. | | |
| When such thoughts arise, they can really affect you, you know. You might feel foolish or overwhelmed. Sometimes, these thoughts can lead a person to seem confused, but in reality, they have their own thoughts and concerns. | | |
